# Epidemiology of soil-transmitted helminth infections in Semarang, Central Java, Indonesia

**DOI:** 10.1371/journal.pntd.0008907

**Published:** 2020-12-28

**Authors:** Johanna Kurscheid, Budi Laksono, M. J. Park, Archie C. A. Clements, Ross Sadler, James S. McCarthy, Susana V. Nery, Ricardo Soares-Magalhaes, Kate Halton, Suharyo Hadisaputro, Alice Richardson, Léa Indjein, Kinley Wangdi, Donald E. Stewart, Darren J. Gray

**Affiliations:** 1 Department of Global Health, Research School of Population Health, Australian National University, Acton, Australia; 2 Yayasan Wahana Bakti Sejatera Foundation (YWBS), Semarang, Indonesia; 3 Department of Nursing, College of Nursing, Konyang University, Daejeon, South Korea; 4 Faculty of Health Sciences, Curtin University, Bentley, Australia; 5 School of Public Health, Griffith Health, Griffith University, South Brisbane, Australia; 6 QIMR Berghofer Medical Research Institute, Herston, Australia; 7 Public Health Interventions Group, Kirby Institute, University of New South Wales, Kensington, Australia; 8 School of Veterinary Science, University of Queensland, Brisbane, Australia; 9 Institute for Health and Biomedical Innovation, Queensland University of Technology, Brisbane, Australia; 10 Politeknik Kesehatan Kemenkes, Semarang, Indonesia; 11 Statistical Consulting Unit, Australian National University, Acton, Australia; 12 School of Veterinary Science, University of Queensland, Gatton, Australia; 13 School of Medicine, Griffith Health, Griffith University, South Brisbane, Australia; Seoul National University College of Medicine, REPUBLIC OF KOREA

## Abstract

Soil-transmitted helminth (STH) infections are endemic in Indonesia. However, prevalence data for many parts of the country are incomplete. The aim of this study was to determine human STH prevalence and knowledge and practices relating to STH risk behaviour, to provide a current view of the status of STH infection in rural communities in Central Java. A cross-sectional survey of 16 villages was conducted in Semarang, Central Java in 2015. Demographic and household data together with information about knowledge and practices relating to STH and hygiene were elicited through face-to-face interviews. Stool samples were collected and examined using the flotation method. Children (aged 2–12 years) also had their haemoglobin (Hb) levels, height and weight data collected, and BMI estimated. Data were analysed using univariate logistic regression analysis. A total of 6,466 individuals with a mean age of 33.5 years (range: 2–93) from 2,195 households were interviewed. The overall prevalence of STH was 33.8% with *Ascaris lumbricoides* (roundworm) the predominant nematode identified (prevalence = 26.0%). Hookworm and *Trichuris trichiura* (whipworm) were found in 7.9% and 1.8% of participants, respectively. Females were at increased odds of infection with *A*. *lumbricoides* (adjusted OR 1.14, 95% CI [1.02–1.29], p = 0.02). Adults in age groups 51–60 and over 60 years had the highest odds of being infected with hookworm (adjusted OR 3.01, 95% CI [1.84–4.91], p<0.001 and adjusted OR 3.79, 95% CI [2.30–6.26], p<0.001, respectively) compared to 6–12 year olds. Farmers also had higher odds of being infected with hookworm (adjusted OR 2.36, 95% CI [1.17–4.76], p = 0.02) compared to other occupation categories. Poverty (OR 2.14, 95% CI [1.77–2.58], p<0.001), overcrowding (OR 1.35, 95% CI [1.27–1.44], p<0.001), goat ownership (OR 1.61, 95% CI [1.10–2.41], p = 0.02) and the presence of dry floor space in the home (OR 0.73, 95% CI [0.58–0.91], p = 0.01) were all household factors significantly associated with an increased odds of infection. Infection with STH was not significantly associated with the gastrointestinal illness (p>0.05), BMI or Hb levels; however, one third of all 2–12 year olds surveyed were found to be anaemic (i.e. Hb concentrations below 110g/l or 115g/l for children under 5 and 5 years or older, respectively), with a greater proportion of school-age children at risk. Knowledge and behaviour related to hygiene and gastrointestinal diseases varied widely and were generally not associated with STH infection. The study revealed that STH infection remains endemic in Central Java despite ongoing deworming programs. Current control efforts would benefit from being re-evaluated to determine a more effective way forward.

## Author summary

Among the major NTDs, STH are one of the most common disabling chronic infections. Currently available drug treatments, whilst considered safe and generally well tolerated, do not confer protection against new infections. In Indonesia, prevalences of STH of up to 90% have been reported but these estimates are based on data from the 1980s and 90s. More up-to-date STH prevalence estimates are urgently needed to help guide future control efforts.

A cross-sectional survey was conducted in rural villages in Semarang, Central Java to determine human STH prevalence and associated risk factors. One-third of all cohort participants were positive for STH with prevalences of 26%, 7.9% and 1.8% identified for *Ascaris lumbricoides*, hookworm and *Trichuris trichiura* at 7.9% and 1.8%, respectively. Risk of *A*. *lumbricoides* infection was higher for females, whilst farmers and adults over 50 had an increased risk of hookworm infection. Poverty, overcrowding in the home and goat ownership were also associated with an increased risk at the household level.

Soil-transmitted helminthiases remains a significant health problem in Central Java, Indonesia, exacerbated by limited knowledge about STH, poor sanitation and hygiene and poverty prevalent in the region. Control efforts would benefit from an integrated approach emphasising WASH, health education and chemotherapy. Further studies investigating environmental contamination with STH in and around homes in endemic areas could provide further insight into links between household factors and STH identified in our study.

## Introduction

Soil-transmitted helminths (STH) are a group of intestinal parasites most commonly referring to *Ascaris lumbricoides* (roundworm), *Trichuris trichiura* (whipworm) and the hookworm species, *Necator americanus* and *Ancylostoma* spp. (including *A*. *duodenale* and *A*. *ceylanicum*) [1]. It is estimated that nearly one quarter of the world’s population is infected with STH [2], accounting for a loss of 5.2 million DALYs [3]. Infections with STH occur primarily in the tropical and subtropical regions of sub-Saharan Africa, the Americas and Asia where the warm moist environments favour worm egg and larvae survival, socioeconomic risk factors such as poor hygiene and sanitation prevail, and where limited access to safe water sources facilitates transmission [1]. Children, in particular, have a high risk of infection and it is estimated that more than 270 million preschool-aged and 550 million school-aged children are infected with STH [4]. Infection in children can lead to physical, nutritional and cognitive impairment affecting their schooling and perpetuating the cycle of poverty [5].

Indonesia is particularly vulnerable to STH infections due to ideal environmental and socioeconomic conditions in many areas [6,7]. Nearly 200 million people across 31 provinces are estimated to be at risk of STH infection [6]. Parasitological surveys carried out in the 1980s and ‘90s estimated the prevalence of *A*. *lumbricoides*, *T*. *trichiura* and hookworm to range from 14–90%, 1–91% and 21–89% respectively [8]. In Semarang, Central Java, the location of this study, STH prevalence ranged from 20–50% [3,9] but these estimates are based on data that are more than a decade old.

Treatment for STH infection with one of the anthelmintic drugs, mebendazole or albendazole is inexpensive and therapy is generally well tolerated. Efficacy varies across the STH species: both drugs are considered highly effective for *A*. *lumbricoides* (up to 100%) but less effective for hookworm and *T*. *trichiura*. The current STH control strategy undertaken in Indonesia is to provide chemotherapy (single 400 mg dose of albendazole) on a yearly basis in areas where STH prevalence is between 20–50% and biannually where prevalence exceeds 50%, as per WHO recommendations [7]. Due to budget constraints however, the frequency and coverage of chemotherapy does not always meet set guidelines, meaning treatment may only occur bi-annually and not be distributed in all at-risk areas. Furthermore, treatment does not prevent re-infection and concerns have been raised over reducing efficacy and potential parasite resistance to the main chemotherapeutic drugs [10].

Prevention and control of STH requires an integrated approach that includes improving sanitation and hygiene behaviour. The aim of this study was to estimate human STH prevalence and the level of knowledge and practices relating to STH (and other gastrointestinal infection) risk. The surveys reported here were conducted within the context of informing a subsequent latrine intervention trial in the region.

## Materials and methods

### Ethics statement

This study was carried out in accordance with relevant guidelines, and written informed consent was obtained from all participants, including the parents or guardians of all child participants. Ethics approval was obtained from Griffith University’s Human Research Ethics Committee (PBH/17/11/HREC) and from Diponegoro University (068/EC/FK- RSDK/2014).

### Study setting

The study was undertaken in two sub-districts–Gunungpati and Mijen–of Semarang City, Central Java. Semarang City is the capital of Central Java, and the fifth most populated city in Indonesia (approximately 1.8 million) [11]. Elevation ranges from two metres below sea level up to 340m [12]. Villages surrounding Semarang City have a large rural population where a substantial proportion of village households do not have latrines and open defecation is common [13].

### Study design

A cross-sectional survey of 16 villages was undertaken to determine individual, household and village-level prevalence of STH, and knowledge and practices relating to water, sanitation and hygiene (WASH) behaviour. Households were randomly selected and all individuals residing in the house who met the inclusion criteria were recruited until a maximum cohort of 550 people per village was reached. Inclusion criteria were as follows: aged at least 2 years; deemed to be physically and mentally competent to answer the interview questions by the interviewer; agreed to participate in the study; and provided informed consent.

### Data collection

Two structured questionnaires (adult and child) were developed in English (S1 Text and S2 Text) and translated to Bahasa Indonesia. The adult questionnaire (for participants aged 13 years and above) was comprised of 9 sections: demographics, housing conditions, latrine use, water access and usage, hand washing practices, keeping of animals, knowledge and behaviours associated with gastro-intestinal and helminth-related diseases, together with a section for interviewers to record the condition of respondent’s hands and nails (i.e. nail biting and cleanliness). Respondents were also asked if they had experienced bowel issues in the previous 3 months such as diarrhoea (with or without abdominal pain), dysentery or recurrent typhoid. The child questionnaire (for respondents aged 2–12 years) was identical with the exception that the sections on housing conditions and animals were omitted. Children were also not asked questions on water usage beyond the source of water for drinking. Parents of children below the age of five were asked to answer on behalf of their child.

Questionnaires were administered by face-to-face interviews in the period between February and April 2014. Interviews were conducted by a team of nurses, midwives and public-health workers who had undergone training in the practice of sample collection and interviewing, to ensure the purpose and meaning of each question was clear as well as to ensure they were able to identify individuals who meet the inclusion criteria.

All children aged 2–12 years in the selected cohort in each village were also asked to have their haemoglobin concentration (Hb) checked via finger prick test. Haemoglobin readings were not adjusted for altitude. Biometric (height, weight) data were also collected and BMI estimated. Calculations and terminology for BMI were based on Barlow SE and the Expert Committee [14]. Calculations for anaemia were based on WHO definitions [15].

All respondents received a plastic container for faecal samples, labelled with their unique personal identification number (PID), and the date of distribution. All participants were instructed to deposit samples from their own faeces into the container and to return the container immediately to one of several collection points. An aliquot (approximately 2g) of each sample was then transferred to a 10 mL plastic centrifuge tube, fixed with 10% formalin and stored at room temperature.

### Stool sample processing

Stool samples were examined using the faecal floatation method outlined in [16] to assess STH infection status with the modification that the presence or absence of eggs were recorded rather than counting the number of eggs.

Quality control was undertaken by independent expert microscopists from Department of Medical Parasitology, Diponegoro University, on 10% of the samples.

### Data management

Data were recorded on specifically designed paper forms. Data were double entered into an MS Access database in English. Electronic copies of entered data were saved offline with paper duplicates and stored in a secure fireproof location for backup. Workshops on the principles of data management and specific database training were delivered to local Indonesian staff for capacity building, and to ensure standardised database use.

### Data analysis

#### Knowledge and behaviour score analysis

Knowledge and behaviour were assessed using a scoring system based on the number of correct responses. Knowledge scores were based on 18 questions (maximum 25 points) relating to gastrointestinal and helminth-related disease transmission, symptoms and prevention. Behaviour scores were based on 10 questions (maximum 41 points) relating to handwashing and other hygiene practices. All questions with yes or no responses were allocated a maximum of 1 point each for correct answers. Questions with scale-like responses were graded with optimal answers allocated the highest score of 3 and least desirable answers given zero points. For example, if a respondent reports “always” washing their hands before eating they would be allocated the maximum number of points (i.e. 3), “often” would receive 2 points, “sometimes” with 1 point and “never” with 0 points. For all questions, no points were allocated for “don’t know” or where no response was provided. Henceforward, the term ‘score’ refers to scores as a percentage of the maximum number of points.

#### Income and poverty calculations and definitions

US dollar conversions for household incomes were calculated based on the average exchange rate in 2014 ($1 USD = 11, 864.40 IDR) [17]. Household poverty index was calculated by dividing daily household incomes in USD [i.e. (monthly income x 12)/ 365)] by the number of household members. Households that fell below the World Bank definition of poverty [18], which prior to 2015 was set at $1.25 USD/person/day, were categorised as ‘below’ and any household with income greater than this amount fell into the ‘above’ group.

### Statistical analyses

Infection prevalence was determined at the individual, household and village level. A household was considered positive if at least one cohort member was positive for at least one species of STH. Village prevalence was calculated by the number of positive households to account for the hierarchical nature of the sampling method.

Descriptive statistics were used to identify trends and patterns in the data. Statistical analyses were applied to determine whether STH infection status was significantly associated with sociodemographic or household variables (i.e. independent groups) at the individual and household level. Pearson’s chi-squared (χ^2^) test and the Welch Two Sample t-test were used to compare mean prevalence of STH infection between independent groups. In cases where cell counts were small, Pearson’s χ^2^ test with simulated p-value (based on 2000 replicates) was used. Spearman’s Rho rank correlation was used to determine whether knowledge and behaviour scores were correlated.

Univariate binomial logistic regression models were used to estimate the odds of STH infection between independent groups. Odds ratios and 95% confidence intervals (CI) were calculated and reported for all models. Due to the hierarchical nature of the data, random intercepts were included accounting for clustering at the household level where prevalence at the individual level was measured. Effects of household factors on STH infection were determined at the household level. Adults and children were included in all models. A P-value of 0.05 was used as the measure of statistical significance in all analyses. All statistical analyses were conducted in R version 3.4.0 (R Core Team, 2017) and Microsoft Excel.

## Results

### Cohort demographic and household data

A total of 6,466 individuals from 2,195 households (mean number of participants per household = 2.95, SD = 1.48), across 16 villages, were surveyed with equal distributions of males and females (49.9% vs. 49.6%). The cohort consisted of 5,305 adults (aged 13–93 years, mean = 39.3 years) and 1161 children (aged 2–12 years, mean = 7.0 years) with an overall mean age of 33.5 years (SD = 19.9). Among the adult cohort who provided employment details and were not students at the time of interview (n = 4664), more than half (n = 2573) had not been educated beyond junior school level. The least educated were the farmers (0.6% with college or higher qualifications) and the highest were the public (56.7%) and private sector (41.4%) employees.

Demographic profiles of study villages are provided in S1 Table. Statistically significant differences were identified in the sample population across villages in mean age (p = 0.002), education levels (p<0.001) and employment categories (p<0.001).

Of the 2,195 households surveyed, data on household characteristics were collected from 2,163 (98.5%). Households typically had four members (mean = 3.67, SE = 0.03) and an average monthly income of IDR 1.1M (range: IDR 25,000 – 14M), equivalent to approximately $96 USD, or $0.79 USD per person per day.

### STH prevalence

One third (n = 2191) of the cohort tested positive for STH. Amongst the entire cohort, *A*. *lumbricoides* was the most common species identified at a prevalence of 26.0% (n = 1,682), followed by hookworm (7.9%) and *T*. *trichiura* (1.8%). Hookworm infection was slightly more common among adults compared to children (8.5 vs. 5.0%, respectively, p<0.001) but no significant difference in prevalence between adults and children was identified for STH in general (34.1 vs. 32.7%, respectively, p = 0.38), *A*. *lumbricoides* (25.7 vs. 27.7%, p = 0.5) or *T*. *trichiura* (1.8 vs. 1.7%, p = 0.90). Polyparasitism was identified in 5.6% (n = 116) of infected individuals with no significant difference between adults and children (1.8 vs. 1.7%, respectively, p = 0.94). Three individuals, all from the adult group, were positive for all three species.

**Fig 1:**
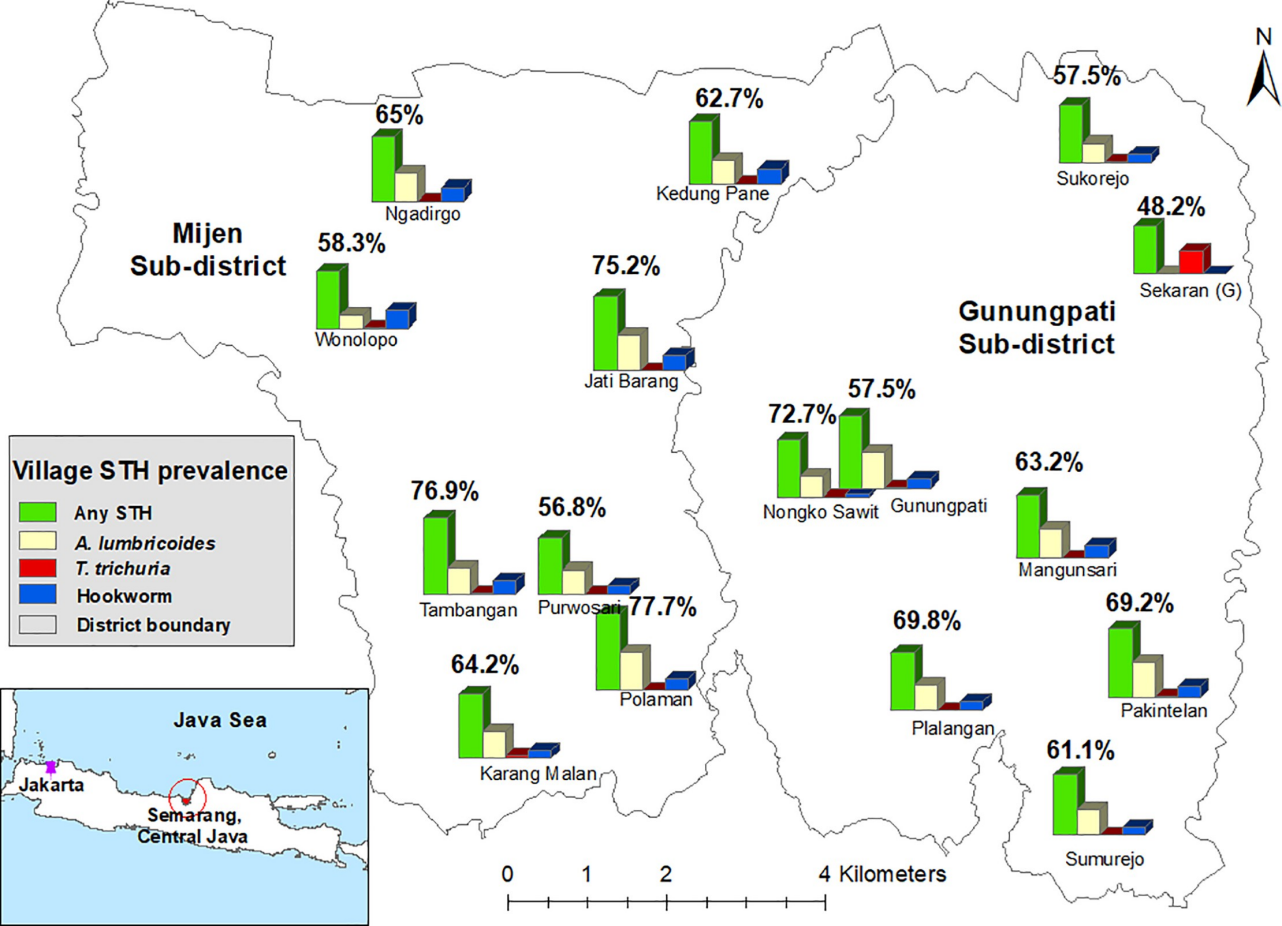
Prevalence of soil-transmitted helminth infected households in each surveyed village plotted onto a map of Semarang, Central Java (village locations are approximate). Map created by Kinley Wangdi.

Prevalence of STH infection (as a proportion of households) across surveyed villages are shown in Fig 1 (prevalence and 95% CIs are also provided in S2 Table). The proportion of STH positive households across villages varied between 48.2% and 77.7% with differences found to be statistically significant p<0.001). Hookworm and *A*. *lumbricoides* were each identified in 15 of the 16 surveyed villages with prevalence ranging from 0–18.5% (p<0.001) and 0–38.4% (p<0.001), respectively. For six of the seven villages in which *T*. *trichiura* was identified, infection rates were very similar (0.18–2.9%, p<0.001). One exception was Sekaran village, where the prevalence for *T*. *trichiura* was 22.5%, which was more than 12 times greater than the overall prevalence. Interestingly, Sekaran was the only surveyed village where no *A*. *lumbricoides* infection was identified and only one individual among 569 participants was found to harbour hookworm.

Of the 2163 households sampled, and from which household demographic data were collected, 1373 (63.5%) had at least one infected individual (i.e. positive for any STH). In the 1993 (92.1%) households with >1 household members, 28.3% (564/1993) had multiple infected members and a prevalence of 100% was identified in approximately 10% (197/1993) of households. The number of households positive for *A*. *lumbricoides*, *T*. *trichuria* and hookworm were 1132 (52.3%), 88 (4.1%) and 408 (18.9%), respectively.

### Association between STH infection and individual demographic factors

Table 1 outlines the association between demographic factors and prevalence of STH infection. *T*. *trichuria* infection was not associated with any demographic factors. *A*. *lumbricoides* infection was slightly more common in females (27.27% in females vs. 24.71% in males, p = 0.02) and the odds of infection for females was found to be 14% higher compared to males (adjusted OR 1.14, 95% CI [1.02–1.29], p = 0.02).

**Table 1:** Demographic background of respondents and prevalence of STH infections.

Variable	N (%)	STH prevalence (n = 2191)		*A*. *lumbricoides* prevalence (%) n = 1682		*T*. *trichiura* prevalence (%) n = 117		Hookworm prevalence (%) n = 511	
		n (%)	P-value^a^	n (%)	P-value^a^	n (%)	P-value^a^	n (%)	P-value^a^
Gender									
Female Male	3205 (49.8) 3229 (50.2)	1106 (34.5) 1070 (33.1)	0.26	872 (27.2) 798 (24.7)	0.02	56 (1.7) 60 (1.9)	0.81	241 (7.5) 267 (8.3)	0.29
Age (years) (n = 6444)									
Mean (SD)	33.5 (19.9)	34.3 (20.1)	0.02^b^	33.1 (19.8)	0.31^c^	35.4 (20.3)	0.31^d^	38.7 (21.1)	<0.001^e^
Age group (years) (n = 6444)									
2–5 6–12 13–20 21–30 31–40 41–50 51–60 >60	417 (6.5) 744 (11.5) 903 (14.0) 960 (14.9) 1080 (16.8) 996 (5.5) 732 (11.4) 612 (9.5)	147 (35.3) 233 (31.3) 293 (32.4) 303 (31.6) 380 (35.2) 348 (34.9) 261 (35.7) 220 (35.9)	0.24	121 (29.0) 201 (27.0) 227 (25.1) 232 (24.2) 299 (27.7) 272 (27.3) 177 (24.2) 150 (24.5)	0.27	9 (2.2) 11 (1.5) 10 (1.1) 20 (2.1) 21 (1.9) 17 (1.7) 19 (2.6) 10 (1.6)	0.49	26 (6.2) 32 (4.3) 71 (7.9) 67 (7.0) 75 (6.9) 79 (7.9) 78 (10.7) 80 (13.1)	<0.001
Education–Child (n = 1091)									
Not at school At school	368 (33.7) 723 (66.3)	123 (33.4) 230 (31.8)	0.64	103 (28.0) 195 (27.0)	0.78	8 (2.17) 12 (1.66)	0.72	19 (5.16) 34 (4.70)	0.85
Education–Adult (n = 5122)									
Elementary school Junior school Senior school College or higher	744 2041 1261 1076	271 (36.4) 729 (35.7) 422 (33.5) 335 (31.1)	0.04	181 (24.3) 550 (26.9) 325 (25.8) 260 (24.2)	0.29	722 (2.96) 2003 (1.86) 1244 (1.35) 1057 (1.77)	0.08	91 (12.2) 179 (8.77) 106 (8.41) 64 (5.95)	<0.001
Occupation—Adult (n = 5246)									
Student Private sector Farmer Public sector Self-employed Unemployed Home duties Other/unspecified	582 (11.1) 494 (9.4) 346 (6.7) 54 (1.0) 586 (11.2) 595 (11.3) 869 (16.6) 1720 (32.8)	203 (34.9) 155 (31.4) 141 (40.8) 18 (33.3) 179 (30.5) 209 (35.1) 299 (34.4) 589 (34.2)	0.09	160 (27.5) 109 (22.1) 88 (25.4) 12 (22.2) 140 (23.9) 162 (27.2) 236 (27.2) 440 (25.6)	0.38	8 (1.4) 14 (2.8) 10 (2.9) 1 (1.9) 11 (1.9) 7 (1.2) 16 (1.8) 28 (1.6)	0.36	48 (8.3) 41 (8.3) 52 (15.0) 7 (13.0) 36 (6.1) 54 (9.1) 65 (7.5) 146 (8.5)	0.001

a Chi-square test comparing infected and uninfected groups

b T-test (t = -2.13, df = 4337.3, p = 0.021)

c T-test (t = 1.02, df = 2958.9, p = 0.307)

d T-test (t = -1.02, df = 120.2, p = 0.310)

e T-test (t = -5.84, df = 585.9, p <0.001)

Hookworm infection was associated with age, education and occupation. The odds of hookworm infection were highest among people aged 51–60 (adjusted OR 3.01, 95% CI [1.84–4.91], p<0.001) and over 60 years (adjusted OR 3.79, 95% CI [2.30–6.26], p<0.001) when compared to children aged 6–12 years, the group with the lowest prevalence. People in age groups 13–20 (adjusted OR 1.98, 95% CI [1.22–3.21], p = 0.01), 21–30 (adjusted OR 1.77, 95% CI [1.08–2.88], p = 0.02), 31–40 (adjusted OR 1.80, 95% CI [1.12–2.88], p = 0.01) and 41–50 (adjusted OR 2.19, 95% CI [1.35–3.52], p<0.001) all had approximately twice the odds of hookworm infection compared to the reference group. In the adult group, having a college or higher degree of education reduced the odds of hookworm infection (adjusted OR 0.38, 95% CI [0.22–0.66], p<0.001) and any STH (adjusted OR 0.78, 95% CI [0.63–0.96], p = 0.02), when compared to those who had completed elementary school. Hookworm infection was highest among farmers (15.0%) and public sector workers, i.e. government officers or military personnel, (13.0%) and lowest among the self-employed (6.1%). Differences in prevalence between occupation groups were statistically significant (p<0.001). Univariate logistic regression analysis revealed that the odds of being infected with hookworm was more than two times higher for farmers (adjusted OR 2.36, 95% CI [1.17–4.76], p = 0.02) compared to unemployed cohort members. Whilst being self-employed acted as a protective factor against infection (adjusted OR 0.41, 95% CI [0.20–0.85], p = 0.02).

### Association between STH infection and household characteristics

Table 2 outlines the association of STH infection and household characteristics. Households positive for STH infection (i.e. at least one infected member) were more likely to be living below the poverty line (70.1% vs. 52.4% for below and above the poverty line, respectively, p<0.001) and had on average more residents compared to STH negative households (3.9 [SE = 0.04] vs. 3.2 [SE = 0.06], p<0.001). Prevalence of STH among households was also higher in households with goats (p = 0.02) and those which reported having dry/sealed floors in at least one area of the house (p = 0.01), and there was an increasing trend in prevalence as household crowding (i.e. the number of people/m^2^) values increased (p<0.001).

**Table 2:** Association between household characteristics and household STH infection status (n = 2163).

Household characteristics	STH prevalence (%)	P-value^a^
Household wealth index^b^ (n = 100 missing values) Below poverty line (n = 1383) Above poverty line (n = 680)	70.1 52.4	<0.001
Dry latrine floor (n = 214) No dry latrine floor (n = 1949)	61.7 63.7	0.62
Dry floor space in the house (n = 380) No dry floor space in house (n = 1783)	64.8 57.4	0.01
Wall material of house 100% bamboo (n = 12) 100% wood (n = 389) 100% brick (n = 1307) 100% brick & other (n = 407) Other/mixed (n = 22)	58.3 62.2 64.8 60.7 50.0	0.34^c^
Household with latrine (n = 995) Household without latrine (n = 1168)	65.0 62.2	0.18
Household latrine located inside home (n = 877) Household latrine located outside home (n = 104)	64.9 65.4	1.00
Animals in household (n = 911) No animals in household (n = 1252)	65.8 61.8	0.07
Goats in household (n = 134) No goats in household (n = 2029)	73.1 62.8	0.02

a Chi-squared test

b Based on $1.25USD/person/day using 2014 average exchange rate

c Chi-squared test with simulated p-values

Univariate analyses revealed that the odds of a household being positive for STH increased by 35% (OR 1.35, 95% CI [1.27–1.44], p<0.001) for every additional person living in a household. Households with goats had 61% (OR 1.61, 95% CI [1.10–2.41], p = 0.02) higher odds of being STH positive compared to households without goats. Yet there were no significant differences in STH prevalence for households with or without cats (51.1% vs. 63.7%, respectively, p = 0.11), dogs (50.0% vs. 63.5%, respectively, p = 0.62), cows (67.4% vs. 63.4, p = 0.70), chicken (65.9% vs. 62.3%, p = 0.11), ducks (76.2% vs. 63.2%, p = 0.12) or geese (83.3% vs 63.4%, p = 0.24). There was also no association between STH infection and ownership of animals in general (65.8% vs. 81.6%, p = 0.07). Further investigation into households with goats compared to those without goats found no identifiable differences in the proportion of households below the poverty index (71.2% vs. 66.8%, respectively, p = 0.04) or those with a latrine in the house (47.7% vs 45.9%, respectively, p = 0.74). There was however a borderline significant difference in the number of household members with and without goats (3.25 vs. 2.95, respectively, t = 1.98, df = 145.8, p = 0.049).

Interestingly, the absence of dry/sealed floors in the house decreased the risk of infection (OR 0.73, 95% CI [0.58–0.91], p = 0.01) compared to households with dry spaces. Yet the amount of space covered in sealed/dry floors in the home (i.e. 25%, 25–50%, 75% or 100% coverage) did not influence whether a household was STH positive or not (p = 0.06)). Most strikingly, the odds of a household being positive for STH infection was more than two-fold greater for households with income levels below the international poverty line (OR 2.14, 95% CI [1.77–2.58], p<0.001) compared to households above the poverty line.

The vast majority (85.8%) of participants reported a single source of drinking water of which, artesian source (30.6%) and household well (29.7%) were the most common. Nearly all (94.6%) adults surveyed reportedly boil water prior to drinking and 85.6% of those who solely drink water that is bottled (n = 344) check to see if the seal is intact. There was no association between source of drinking water and STH infection (p = 0.78).

### Association between STH infection and morbidity measures

Gastrointestinal illness in the previous three months was reported by 4.6% (n = 294) of the cohort population (n = 85 missing values) with a mean of 2.1 episodes (SD = 1.5). Neither the presence nor frequency of gastrointestinal illness were statistically associated with STH infection (p = 0.62 and p = 0.50, respectively).

In children aged 2–12 years, there were no significant differences in height, weight, BMI and blood haemoglobin (Hb) levels between STH infected and uninfected individuals (S3 Table). However, one third (n = 292) of all children surveyed fell within mild to severe anaemic ranges with a greater proportion of school-aged children suffering some level of anaemia compared to non-school aged (34.7% vs. 29.2, p<0.001) (S4 Table). Differences in BMI category were also observed, with obesity levels among non-school aged children double that of school-aged children (20.2 vs. 9.6%, p < .001) (S5 Table), with the latter more likely to be of normal BMI (61.6 vs. 51.8%).

### Gastrointestinal and helminth-related disease knowledge

Knowledge of gastrointestinal and helminth-related disease was limited as indicated by the low mean score of 54.7% (SD = 13.3). Overall, knowledge of the causes of bowel infections was lowest with only seven (0.12%) respondents correctly identifying worms, bacteria or viruses, which is less than the number (n = 11) who believed witchcraft or Satan were responsible. Less than 10% (n = 612) were aware that worms can cause illness and 84% and 98% of the cohort were unable to identify a single symptom of gastrointestinal parasite infection, respectively. Awareness of the potential for domestic animals to transmit disease and possibility of human faeces containing bacteria and viruses was higher (78.3% and 63.9%, respectively). Similarly, the vast majority (89.7%) of participants were aware that keeping domestic animals in a separate enclosure and people not defecating in rivers or garden beds (89.2%) are considered good practice to prevent transmission of infectious diseases.

### Hygiene and sanitation practices related to gastrointestinal illness and worms

Less than half (n = 3,133) of the cohort population reported having a latrine in their home. The use of water, either in the bathroom (65.7%) or from the river (18.2%), was the preferred method of cleaning oneself after a bowel motion. The presence of a latrine in the family home was not associated with STH infection (p = 0.52). Cleaning practices after having a bowel motion were also not associated with STH infection (p = 0.14). Additional details on latrine usage and cleaning practices are provided in S6 Table.

Handwashing was most often practiced before prayers (71.8%), eating (61.3%) and food preparation (57.3%) (S7 Table). Only half of the cohort reported always washing their hands after using the toilet, similar to that reported for when coming home (48.8%). Using soap every time during handwashing was reported by 57% (n = 3578) of cohort participants. The main reasons reported by participants (n = 2599 responses) for not using soap during handwashing were forgetting (33.4%), habit (25.0%), being in a hurry (21.0%) and no soap available (19.8%). Soap use was not found to be significantly associated STH infection (p = 0.27).

Visiting paddy or other fields was the only practice associated with STH infection (S8 Table), with a lower risk seen among participants who never visit paddy fields (OR 0.67, 95% CI [0.55–0.82], p<0.001) or only on a monthly basis (OR 0.78, 95% CI [0.61–1.00], p = 0.046) compared to daily visitors.

Behaviour scores ranged from 7–100% (mean = 67.6, SD = 15.0). No association was identified between behaviour score and STH infection (t = -0.09, df = 4349, p = 0.93). Behaviour and knowledge scores were moderately, positively correlated (rho: 0.42, p<0.001).

## Discussion

Our findings indicate STH infections are ubiquitous throughout this region of Central Java given that all 16 villages and 63% of households surveyed were positive for at least one STH species. Although the overall prevalence of 33% identified in this study is below infection levels reported from the late 1990s [19], it is likely to be an underestimate of the true prevalence as identified by molecular diagnostic techniques (i.e. qPCR) which are far superior in sensitivity [16,20], particularly for hookworm infections [21]. Despite the higher sensitivity and other benefits of using molecular diagnostic techniques (e.g. the ability to detect multiple species in a single assay) [22], the cost of the highly specialised equipment, kits and reagents limits the feasibility in remote or low-resource settings.

Notable differences were identified in the prevalence of STH infection across villages that we were unable to explain based on the variables assessed in this study. Several environmental factors have been associated with STH prevalence and intensity of infection. High precipitation, sandy-loam soil, high vegetation index and higher temperatures have been associated with increased hookworm prevalence and infection intensity [23–25]. In contrast, *A*. *lumbricoides* and *T*. *trichiura* infection have been negatively associated with higher rainfall and temperatures, heavily wooded areas and alkaline, clay-loam soils [23,25–28]. Given the pattern of worm distribution was comparable among the vast majority of study sites, it is unlikely that environmental factors played a significant role in explaining the differences in STH prevalence between villages. However, this cannot be ruled out, especially as our study did not assess environmental variables which may have identified key similarities or differences between study sites. The high levels of *T*. *trichiura* and absence of *A*. *lumbricoides* identified in Sekaran could also not be explained by demographic or household factors. Sekaran was however, among four villages where goat ownership was more common and in our study, keeping goats raised the household odds for STH infection by 61%. Although goats can be infected with a number of helminth species [29–32], there have been no studies identifying goats as a source of human STH infection and the finding might relate to unidentified confounding, such as Type 1 statistical error or other household and environmental factors not explored in the current study. Nonetheless, as there were no clear identifiable differences between villages that might provide a plausible explanation for the comparatively high levels of *T*. *trichiura* in Sekaran or the vastly differing prevalences, further investigation is warranted.

Prevalence for STH infections often exhibit age-dependent patterns, with highest levels typically seen in early childhood and decreasing into adulthood. This is particularly the case for *A*. *lumbricoides* and *T*. *trichiura*, where peak prevalence is generally seen in children under the age of five years [5]. Although we observed a similar pattern in our study, the differences were not statistically significant. In contrast, hookworm infection usually exhibits peak prevalence in adulthood [10]; an observation which our study findings also support as indicated by the positive correlation between risk of infection and age.

It is well established that people engaging in agricultural work are at a greater risk of hookworm infection due to frequent and extended exposure to potentially contaminated soil compared to non-farmers or agricultural workers [33]. We also found that education had a protective effect for hookworm infection, and STH in general, and the farmers in our study were less educated compared to other occupations, possibly compounding their risk. Based on these findings, farmers are a key demographic who would benefit from targeted chemotherapy and health education interventions.

Although *A*. *lumbricoides* infection dominated in our study, we were unable to identify any meaningful risk factors beyond being female, which only marginally increased the risk of infection. Gender differences have been reported in other studies [25,34,35] and have generally been attributed to differences in day-to-day activities but typically, *A*. *lumbricoides* infection is not associated with one gender or the other.

Poverty is often reported to be the most significant risk factor for STH infection and our findings provide further evidence to support this link. According to the World Bank’s definition of poverty, two-thirds of households in our study fell below the extreme poverty level (less than $1.25 USD per person, per day) and we found the risk of infection to be two-fold greater in households living below the international poverty line compared to those above. Limited access to safe drinking water, poor sanitation and overcrowding in homes are all common themes of poverty. Although we found no link between STH infection and drinking water sources or with the presence of latrines in the family home, overcrowding was common in our cohort, and similar to other studies [36–38], was associated with an increased risk for STH infection at the household level. Horizontal transfer or focal transmission (e.g. from sharing a contaminated environment) among family members within close proximity to the home may explain this observation [39]. The structure and materials used for dwellings are also indicators of wealth and have previously been linked to higher prevalence of STH infection [33]. In our study, housing materials (e.g. wood, bamboo or brick) were not associated with STH, yet the presence of dry floor space inside a dwelling was found to influence the risk of a household being infected. Contradictory to that which is typically reported [33,39], our results indicate that the risk was greater for homes with at least one area of the house covered in dry/sealed floors. This may be related to the environmental stability of different helminth species as suggested in a recent study conducted in rural Bangladesh which found cement floors only reduced the risk for *A*. *lumbricoides* but not for *T*. *trichiura* or hookworm [40]. However given that in our study, *A*. *lumbricoides* was the most common worm type identified and the amount of dry/sealed floor space (i.e. whether 25%, 25–50%, 75% or 100% of the home) did not influence STH infection status of the household, it is more likely that our finding represents an incidental link. Further investigation into environmental contamination in and around homes in our study area would provide more insight into possible explanations for the associations identified and reported here.

Although anaemia could not be linked to STH infection in this study, possibly due to low prevalence and intensity of hookworm infection, we did observe a high prevalence of anaemia in children, especially those of school age. Compared to 2007/2008 levels reported by Barkley et al. [41], our findings suggest the prevalence of anaemia among pre-school aged children (2–5 years) has remained the same (29.2 vs. 31.4%, respectively) and increased among school-aged children (34.7 vs. 20.6%). These findings indicate anaemia is an ongoing health problem in children and identifying the causes and treating cases should be a high priority for local health authorities. The lack of an association between STH infection and health measures including anaemia, BMI and diarrhoeal illness (in adults and children) in this study could indicate light infections, as mentioned above, and/or a nutritional deficit within the communities as a whole [42]. It is also possible that our sample size was insufficient to detect associations between these variables. Nonetheless, our findings add to the growing list of studies in recent years failing to identify a link between STH infection and morbidity measures [43–45].

Transmission of *A*. *lumbricoides* and *T*. *trichiura* occurs via the faecal-oral route, meaning that infection typically results from the ingestion of infective eggs either through contaminated food, water, eating utensils or sucking fingers or biting fingernails of improperly washed hands. However, we failed to find a link between STH infection and WASH practices that may put an individual at risk of infection. Several recent studies have also failed to demonstrate an association between WASH and STH infection [23,46–48], even though WASH has been proposed as an important intervention to reduce the risk of infection [2]. Relying on self-reported behaviours can be problematic given that respondents may not always be accurate in their responses [49].

The high levels of poverty and limited knowledge related to STH identified in our study, in combination with ideal environmental conditions and inconsistency in preventative chemotherapy may be facilitating the perpetuation of STH in the environment. The resulting high levels of environmental contamination suggest that even the most rigorous hygiene practices may not be sufficient to limit exposure to infective eggs. Further studies investigating the level of environmental contamination would be highly beneficial in these locations.

Our study was limited by several factors, which may have influenced our findings and interpretations. Firstly, anthropometric data were only collected for children aged 2–12 years and the relatively small number of children included in the study meant that we were unable to assess whether BMI and haemoglobin levels were associated with *A*. *lumbricoides*, *T*. *trichiura* or hookworm infection individually. Secondly, we did not measure intensity of infection, which may have revealed further insight into the absence of a link between health measures, including hookworm associated anaemia or the presence of gastrointestinal symptoms such as diarrhoea that are more common in higher intensity and in *T*. *trichiura* infections [50]. Due to the low prevalence of *T*. *trichiura* and hookworm, the egg counts were often too small to extract any statistically significant findings and therefore analyses for health measures, behaviours and knowledge were assessed only at the overall STH infection level thus making it difficult to identify risk factors for these STH species.

In conclusion, findings from this study demonstrate that STH infection is still prevalent in Semarang, Central Java despite ongoing deworming programs. Current control efforts in the region are largely focused on mass drug administration rather than an integrated approach that also emphasises WASH and health education, and chemotherapy does not always meet set guidelines. This combined with limited knowledge about causes and prevention of gastrointestinal and helminth-related disease, poor hygiene and sanitation practices, poverty and ideal environmental conditions facilitating STH transmission may be contributing factors as to why STH infections persist in the region. Current control efforts would benefit from being re-evaluated to determine a more effective way forward.

## Supporting information

S1 Text Adult—Helminth Education and Latrine Project (HELP) Questionnaire. (DOCX)

S2 Text Child—Helminth Education and Latrine Project (HELP) Questionnaire. (DOCX)

S1 Table Participant and selected household demographics for each surveyed village in Semarang. (DOCX)

S2 Table Village level prevalence of soil-transmitted helminths. (DOCX)

S3 Table STH infection and health measurements for children 12 years and younger. (DOCX)

S4 Table Anaemia in surveyed children between 2 and 12 years old. (DOCX)

S5 Table BMI categories adjusted for age and gender. (DOCX)

S6 Table Latrine usage and cleaning practices. (DOCX)

S7 Table Handwashing practices. (DOCX)

S8 Table Behaviour related to gastrointestinal diseases and worms. (DOCX)
