# Supplementary material for: Epidemiology of soil-transmitted helminth infections in Semarang, Central Java, Indonesia
Source: PLoS Negl Trop Dis. 2020 Dec 28;14(12):e0008907. doi: 10.1371/journal.pntd.0008907 (PMC7793285; doi:10.1371/journal.pntd.0008907)
Supplement: S1 Text — (DOCX) [file pntd.0008907.s001.docx]

|  | d | d |  | m | m |  | y | y | y | y |  |  |
| --- | --- | --- | --- | --- | --- | --- | --- | --- | --- | --- | --- | --- |
| Date: |  |  | / |  |  | / | 2 | 0 | 1 |  | Researcher’s name |  |

**Consent checklist**

| *Obtain written consent - signed letter* Y |  | N |  | ***Only proceed if ‘Yes’*** |
| --- | --- | --- | --- | --- |

***FIELD WORKERS****:* ***YOU WILL COMPLETE THIS QUESTIONNAIRE BASED ON RESPONDENTS ANSWERS***

***Note****: THIS IS ONLY FOR* ***ADULT*** *RESPONDENTS (WHO ARE 12 YEARS OLD OR OLDER).*

*FOR CHILDREN BELOW 12 YEARS OLD, YOU WILL INTERVIEW THE MOTHER/CAREGIVER, BUT* ***BE SURE TO USE THE CHILD QUESTIONNAIRE****.*

**____________________________________________________________________________________**

**ID Information**

Respondent Name: _________________________ __________________________

Family Given

Village Name:­­­­­­­­­­­­­­­­­­­­_________________________________________________

**PID**

| **Sub-district ID**  (1 OR 2) | **Village ID** | **RW** | | **RT** | | **House ID** | | | **Individual ID** | |
| --- | --- | --- | --- | --- | --- | --- | --- | --- | --- | --- |
|  |  |  |  |  |  |  |  |  |  |  |

1. **Demographic Information**

***What is your …? (ask if not easily apparent)***

| 1.1 Gender: Male |  | Female |  |  |
| --- | --- | --- | --- | --- |

| \|  \|  \| \| --- \| --- \|  \|  \|  \| \| --- \| --- \|   1.2 Age: years, | months |
| --- | --- | --- | --- | --- | --- |

1.3 Status within household (✓)

| 1) Head of household |  |  |
| --- | --- | --- |
| 2) Wife |  |  |
| 3) Child |  |  |
| 4) Other family member |  |  |
| 5) Other (not a family member)  *Specify:* | | |

1.4 Highest level of education completed (✓)

| 1) Elementary school |  |
| --- | --- |
| 2) Junior secondary school |  |
| 3) Senior secondary school |  |
| 4) College, or higher |  |

1.5 Employment/ job (✓)

| 1) Company (business) employee |  |
| --- | --- |
| 2) Self-employed, or entrepreneur |  |
| 3) Farmer |  |
| 4) Government officer |  |
| 5) Military |  |
| 6) Other employment |  |
| 7) No employment |  |
| (if 7) a. Home duties |  |
| b. Student |  |
| c. Other  *Specify:* | |

1.6 Household income per month: ___________________Rupiah

1.7 Religion (✓)

| 1) Islam |  |
| --- | --- |
| 2) Catholic |  |
| 3) Protestant |  |
| 4) Buddhist |  |
| 5) Hindu |  |
| 6) Other  *Specify:* | |

**2. Housing conditions**

|  |  |
| --- | --- |

2.1 How many people live in this home? person(s)

|  |  |  |
| --- | --- | --- |

2.2 What is the approximate area of the house? m^2^

2.3 How much floor space is cement/concrete (dry)? (✓)

| Less than 25% | | 25% | | | 50% | | | 75% | | | 100% | | |
| --- | --- | --- | --- | --- | --- | --- | --- | --- | --- | --- | --- | --- | --- |
|  |  | |  |  | |  |  | |  |  | |  |  |

2.4 Which part(s) of the house has a cement/dry floor? (*Multiple responses allowed*) (✓)

| 1) Entrance |  |  |
| --- | --- | --- |
| 2) Bedroom |  |  |
| 3) Kitchen |  |  |
| 4) Guestroom |  |  |
| 5) Latrine |  |  |
| 6) Other  *Specify:* | | |

2.5 What are the walls made of? (*Multiple responses allowed*)

| 1) Bamboo | % |  |
| --- | --- | --- |
| 2) Wood | % |  |
| 3) Brick | % |  |
| 4) Other %  *Specify:* | | |

**3. Use of latrines**

3.1 Where do you usually have a bowel motion? (✓)

| 1) River/bush |  |  |
| --- | --- | --- |
| 2) Public latrines |  |  |
| 3) Neighbours’/relatives’ latrine |  |  |
| 4) Latrine at my home |  |  |
| 5) Other  *Specify:* | | |

3.2 After you have a bowel motion, how do you clean yourself? (✓)

| 1) With leaves |  |  |
| --- | --- | --- |
| 2) In the river |  |  |
| 3) With paper In the bathroom |  |  |
| 4) With water in the bathroom |  |  |
| 5) Other  *Specify:* | | |

3.3 Does your household have a latrine? (✓)

| 1) Yes |  | (✓) *⇒* *If* ***YES***, go to 3.4 below. |
| --- | --- | --- |
|  | | |
| 2) No |  | (✓) *⇒* *If* ***NO***, go to 3.3.1 below. |

3.3.1 *If* ***NO***, why do you not have a family-latrine? (*Multiple responses allowed*) (✓)

| 1) No money |  |  |
| --- | --- | --- |
| 2) No time to build it |  |  |
| 3) Not familiar with toilets |  |  |
| 4) Other  *Specify:* | | |

*⇒* *Now, please* go to 4.1 (page 5).

3.4 Where is your household latrine? (✓)

| 1) Inside the house |  |
| --- | --- |
| 2) Outside the house |  |

3.5 Which kind of latrine is it? (✓)

| 1) Dry only |  |  |
| --- | --- | --- |
| 2) Wet (water closet) only |  |  |
| 3) Sometimes wet, sometimes dry  *Specify when and why*: | | |

**4. Water usage**

4.1 Where do you get water for drinking? (*Multiple responses allowed*) (✓)

|  | ✓ | 4.1.1 If ***YES*** (✓),  how old is it? | 4.1.2 If ***YES*** (✓),  how far is it from a latrine? | |
| --- | --- | --- | --- | --- |
|  |  |  | Distance | No latrine |
| 1) From a public well |  | months | m |  |
| 2) From a household well |  | months | m |  |
| 3) From an artesian source |  | months | m |  |
| 4) From government pipeline (PAM) |  | months | m |  |
| 5) From the river |  | | m |  |
| 6) Buy bottled water |  | | | |
| 7) Other  *Specify:* | | | | |

4.1.3 (If answer to 4.2 was “***Buy bottled water***”) Do you check whether the seal on the bottle is intact? (✓)

| 1) Yes |  |
| --- | --- |
| 2) No |  |

4.2 Do you boil your water for drinking purposes? (✓)

| 1) Yes, always boil water for drinking |  | *⇒* *If* ***YES***, go to 4.2.1 below. |
| --- | --- | --- |
| 2) No, do not always boil water for drinking |  |  |
| 3) Always use and refill bottled water |  |  |

4.2.1 (If answer to 4.2 was “***YES***”) How long do you boil water? (✓)

| 1) Less than 1 minute |  |
| --- | --- |
| 2) Longer than 1 minute |  |

4.3 Where do you get water to wash your kitchen utensils? (*Multiple responses allowed*) (✓)

|  | ✓ | 4.3.1 If YES (✓),  how old is it? | 4.3.2 If YES (✓),  how far is it from a latrine? | |
| --- | --- | --- | --- | --- |
|  |  |  | Distance | No latrine |
| 1) From a public well |  | months | m |  |
| 2) From a household well |  | months | m |  |
| 3) From an artesian source |  | months | m |  |
| 4) From government pipeline (PAM) |  | months | m |  |
| 5) From the river |  | | m |  |
| 6) Buy bottled water |  | | | |
| 7) Other  *Specify:* | | | | |

4.4. Baby’s formula: Where do (did) you get water for your baby’s formula?
 (*Multiple responses allowed*) (✓)

|  | Yes (✓) | 4.4.1 If YES (✓),  how old is it? | 4.4.2 If YES (✓),  how far is it from a latrine? | |
| --- | --- | --- | --- | --- |
|  |  |  | Distance | No latrine |
| 1) From a public well |  | months | m |  |
| 2) From a household well |  | months | m |  |
| 3) From an artesian source |  | months | m |  |
| 4) From government pipeline   (PAM) |  | months | m |  |
| 5) From the river |  | | m |  |
| 6) Buy bottled water |  | | | |
| 7) Never used baby formula |  | | | |
| 8) Other  *Specify:* | | | | |

4.4.3 Do (Did) you boil your water for your baby’s formula? (✓)

| 1) Yes |  | *⇒* *If* ***YES***, go to 4.4.4 below. |
| --- | --- | --- |
| 2) No |  |  |
| 3) Do not know |  |  |

4.4.4 (If answer to 4.4.3was “***YES***”) How long do you boil water? (✓)

| 1) Less than 1 minute |  |
| --- | --- |
| 2) Longer than 1 minute |  |

4.5 Do you use Chlorine tablets in your water? (✓)

| 1) Yes |  |
| --- | --- |
| 2) No |  |
| 3) Do not know about Chlorine tablets |  |

**5. Animals**

5.1 How many animals do you keep? (*Multiple responses allowed*) (✓)

|  | Yes (✓) | 5.1.1 If ***yes,*** how many? |
| --- | --- | --- |
| 1) No animal |  | *⇒ If none, go to 5.3.* |
| 2) Dog |  |  |
| 3) Duck |  |  |
| 4) Goat |  |  |
| 5) Goose |  |  |
| 6) Cow |  |  |
| 7) Cat |  |  |
| 8) Chicken |  |  |
| 9) Other  *Specify:* | | |

5.2 Where are the animals enclosed/caged? (*Multiple responses allowed*) (✓)

5.2.1 Insert type of animal

| 1) No cage/left free |  |  |
| --- | --- | --- |
| 2) In an enclosure inside your house |  |  |
| 3) In an enclosure near your house |  |  |
| 4) In an enclosure some distance from your house |  |  |
| 5) Some left free, some not left free  *Specify*: | |  |

5.3 Can domestic animals transmit disease to people? (✓)

| 1) Yes |  |
| --- | --- |
| 2) No |  |
| 3) Do not know |  |

5.4 To prevent people getting diseases, which is better? (✓)

| 1) Keep domestic animals in a special enclosure |  |
| --- | --- |
| 2) Let animals roam free in the house |  |
| 3) Do not know |  |

**6. Gastrointestinal and helminth-related diseases**

6.1 *Over the past* ***3 months****, have you had a bowel sickness?*
 (*Prompt:* such as diarrhea, abdominal pain with diarrhea, dysentery, recurrent typhoid)

| 1) Yes |  | (✓) How many times? |  |  | *⇒ Go to 6.1.1   (and either 6.1.2 or 6.1.3)* |
| --- | --- | --- | --- | --- | --- |
|  | | | | | |
|  | | | | | |
| 2) No |  | (✓) *⇒ Go to 6.2* | | | |

6.1.1 (*If answer to 6.1 was “****Yes****”*) How did you treat the sickness? (*Multiple responses allowed*) (✓)

| 1) Bought medication at a shop |  |  |
| --- | --- | --- |
| 2) Used public health care centre |  |  |
| 3) Went to see a physician |  |  |
| 4) Was hospitalised as in-patient |  |  |
| 5) Do not remember |  |  |
| 6) No treatment |  |  |
| 7) Other  *Specify:* | | |

6.1.2 (***Adults****: If answer to 6.1 was “****Yes****”*) Were you absent from work due to bowel sickness/infection?

| 1) Yes |  | (✓) How many days?   \|  \|  \| \| --- \| --- \| |
| --- | --- | --- | --- | --- |
|  | | |
| 2) No |  | (✓) |

6.1.3 (***Students****: if answer to 6.1 was “****Yes****”*) Were you absent from school due to bowel sickness/infection?

| 1) Yes |  | (✓) How many days?   \|  \|  \| \| --- \| --- \| |
| --- | --- | --- | --- | --- |
|  | | |
| 2) No |  | (✓) |

6.2 In your opinion, what makes people sick with bowel infections (diarrhea, dysentery, typhoid, etc)?

(*Multiple responses allowed*) (✓)

| 1) Bacteria, or viruses |  |  |
| --- | --- | --- |
| 2) Worms |  |  |
| 3) Do not know |  |  |
| 4) Witchcraft, or Satan |  |  |
| 5) Other  *Specify:* | | |

6.3 Can you help prevent diarrhea by washing your hands before you eat? (✓)

| 1) Yes |  |
| --- | --- |
| 2) No |  |
| 3) Do not know |  |

6.4 Can you help prevent diarrhea by regularly cutting your nails? (✓)

| 1) Yes |  |
| --- | --- |
| 2) No |  |
| 3) Do not know |  |

6.5 Can you help prevent diarrhea by washing eating utensils or kitchen utensils with clean water (boiled
 water, water from sealed bottles, PAM, or artesian source)? (✓)

| 1) Yes |  |
| --- | --- |
| 2) No |  |
| 3) Do not know |  |

6.6 Can you help prevent diarrhea by keeping food away from insects? (✓)

| 1) Yes |  |
| --- | --- |
| 2) No |  |
| 3) Do not know |  |

6.7 Can you help prevent diarrhea by only buying foods that are covered? (✓)

| 1) Yes |  |
| --- | --- |
| 2) No |  |
| 3) Do not know |  |

6.8 Can you help prevent diarrhea by only drinking water that was boiled? (✓)

| 1) Yes |  |
| --- | --- |
| 2) No |  |
| 3) Do not know |  |

6.9 Can you name the types of worms that can be found in the human stomach?
 (*Multiple responses allowed*) (✓)

| 1) Pinworms (Oxyuris) |  |  |
| --- | --- | --- |
| 2) Roundworms (Ascaris) |  |  |
| 3) Whipworms (Ancyclostoma) |  |  |
| 4) Hookworms |  |  |
| 5) Tapeworms |  |  |
| 6) Do not know |  |  |
| 7) Other  *Specify*: | | |

6.10 In your opinion, can worms make you sick? (✓)

| 1) Yes |  |
| --- | --- |
| 2) No |  |
| 3) Do not know |  |

6.11 What are the symptoms of Roundworm infection (Ascaris)? (*Multiple responses allowed*) (✓)

| 1) Fever and dizziness |  |
| --- | --- |
| 2) Coughing up phlegm (sputum) |  |
| 3) Anaemia |  |
| 4) Quickly becoming exhausted |  |
| 5) Do not know |  |

6.12 What are the symptoms of Pinworm infection (Oxyuris)? (*Multiple responses allowed*) (✓)

| 1) Itching in the anus |  |
| --- | --- |
| 2) Abdominal pain |  |
| 3) Do not know |  |

6.13 What are the symptoms of Hookworm infection? (*Multiple responses allowed*) (✓)

| 1) Itching in the anus |  |
| --- | --- |
| 2) Abdominal pain |  |
| 3) Do not know |  |

6.14 Can bacteria and worm eggs be contained in human faeces? (✓)

| 1) Yes |  |
| --- | --- |
| 2) No |  |
| 3) Do not know |  |

6.15 When people pass motions in the river or bush, do you think it can spread those diseases or worms
 we mentioned above? (✓)

| 1) Yes |  |
| --- | --- |
| 2) No |  |
| 3) Do not know |  |

6.16 Do you think the faeces of healthy people can also contain those diseases we mentioned above? (✓)

| 1) Yes |  |
| --- | --- |
| 2) No |  |
| 3) Do not know |  |

6.17 Do you consider that passing a motion in the river or garden is good health behavior or not? (✓)

| 1) Not good |  |
| --- | --- |
| 2) It does not matter |  |
| 3) Good |  |
| 4) Do not know |  |

**7. Washing hands**

7.1 When do you wash your hands? (*Multiple answers possible)*.
 ***Let the participant answer the question and do* not *suggest possible answers.*** ***Place (✓) in the appropriate box below.***

7.1.1 After toilet

| 1) Always |  |  | 2) Often |  |  | 3) Sometimes |  |
| --- | --- | --- | --- | --- | --- | --- | --- |

7.1.2 Before eating

| 1) Always |  |  | 2) Often |  |  | 3) Sometimes |  |
| --- | --- | --- | --- | --- | --- | --- | --- |

7.1.3 After eating

| 1) Always |  |  | 2) Often |  |  | 3) Sometimes |  |
| --- | --- | --- | --- | --- | --- | --- | --- |

7.1.4 Before preparing food

| 1) Always |  |  | 2) Often |  |  | 3) Sometimes |  |
| --- | --- | --- | --- | --- | --- | --- | --- |

7.1.5 After changing diaper

| 1) Always |  |  | 2) Often |  |  | 3) Sometimes |  |
| --- | --- | --- | --- | --- | --- | --- | --- |

7.1.6 When coming home

| 1) Always |  |  | 2) Often |  |  | 3) Sometimes |  |
| --- | --- | --- | --- | --- | --- | --- | --- |

7.1.7 Before prayers

| 1) Always |  |  | 2) Often |  |  | 3) Sometimes |  |
| --- | --- | --- | --- | --- | --- | --- | --- |

7.1.8 Other (Specify:_____________________________________)

| 1) Always |  |  | 2) Often |  |  | 3) Sometimes |  |
| --- | --- | --- | --- | --- | --- | --- | --- |

7.2 How often do you use soap when you wash your hands? (✓)

| 1) Always |  |
| --- | --- |
| 2) Often |  |
| 3) Sometimes |  |
| 4) Never |  |

7.3 Why would you possibly NOT use soap while washing your hands? (✓)

| 1) Forget |  |  |
| --- | --- | --- |
| 2) In hurry |  |  |
| 3) Soap not available |  |  |
| 4) Habit |  |  |
| 5) Other  Specify: | | |

**8. Behaviour related to gastrointestinal diseases and worms**

8.1 Do you go out into the paddy fields or other fields? (✓)

| 1) Always (*every day*) |  |  |
| --- | --- | --- |
| 2) Often (*once a week*) |  |  |
| 3) Sometimes (*once a month*) |  |  |
| 4) Never |  | *⇒ If never, go to 8.3* |

8.2 (***If answered 1)-3) above***) Do you wear shoes/sandals when you go out into the paddy fields or other
 fields? (✓)

| 1) Always |  |
| --- | --- |
| 2) Often |  |
| 3) Sometimes |  |
| 4) Never |  |

8.3 Do you wash or peel fruit before you eat it? (✓)

| 1) Always |  |
| --- | --- |
| 2) Often |  |
| 3) Sometimes |  |
| 4) Never |  |

8.4 Do you eat raw or un-boiled vegetables? (✓)

| 1) Always |  |
| --- | --- |
| 2) Often |  |
| 3) Sometimes |  |
| 4) Never |  |

8.5 Do you eat with a spoon or a similar utensil? (✓)

| 1) Always |  |
| --- | --- |
| 2) Often |  |
| 3) Sometimes |  |
| 4) Never |  |

8.6 How often do you cut your fingernails? (✓)

| 1) Once in a week or more often |  |
| --- | --- |
| 2) About once in two weeks |  |
| 3) Less often than once in two weeks |  |

8.7 Do you bite or suck your fingers/fingernails? (✓)

| 1) Always |  |
| --- | --- |
| 2) Often |  |
| 3) Sometimes |  |
| 4) Never |  |

8.8 Do flies get into your food at home? (✓)

| 1) Always |  |
| --- | --- |
| 2) Often |  |
| 3) Sometimes |  |
| 4) Never |  |

8.9 Do you buy food from street traders if the food is covered? (✓)

| 1) Always |  |
| --- | --- |
| 2) Often |  |
| 3) Sometimes |  |
| 4) Never |  |

**9. Items checked during the visit (observations by interviewer)**

9.1 Can you show me your nails? (✓)

| 1) All clean |  |
| --- | --- |
| 2) Some dirty |  |
| 3) All dirty |  |

9.2 Can you show me your hands? (✓)

| 1) Clean |  |
| --- | --- |
| 2) Somewhat dirty |  |
| 3) Very dirty |  |

9.3 Do you have any itching in your anus today? (✓)

| 1) Yes |  |  | 2) No |  |
| --- | --- | --- | --- | --- |

9.4 Are there worms in your stool today? (✓)

| 1) Yes |  |
| --- | --- |
| 2) No |  |
| 3) Do not know |  |
| 4) No bowel movement today |  |

9.5 Do you have any stomach/abdominal pain at this time? (✓)

| 1) Yes |  |  | 2) No |  |
| --- | --- | --- | --- | --- |

------------------------------------------------------------------------------------------------------------------------

**Thank you very much for participating! I have just one more thing I would like to ask you:**

***Can I measure a number of things about you?***

**10. Anthropometric Information**

|  |  |  |
| --- | --- | --- |

|  |  |  |
| --- | --- | --- |

- 1. Height (cm) 10.2 Weight (kg)
  2. Skinfolds (triceps)_____________(Millimetres)

10.4 Hb________________
