# Supplementary material for: Epidemiology of soil-transmitted helminth infections in Semarang, Central Java, Indonesia
Source: PLoS Negl Trop Dis. 2020 Dec 28;14(12):e0008907. doi: 10.1371/journal.pntd.0008907 (PMC7793285; doi:10.1371/journal.pntd.0008907)
Supplement: S2 Text — (DOCX) [file pntd.0008907.s002.docx]

|  | d | d |  | m | m |  | y | y | y | y |  |  |
| --- | --- | --- | --- | --- | --- | --- | --- | --- | --- | --- | --- | --- |
| Date: |  |  | / |  |  | / | 2 | 0 | 1 |  | Researcher’s name |  |

**Consent checklist: Has written consent been obtained from parent?**

| Yes |  | N |  | ***Only proceed if Yes*** |
| --- | --- | --- | --- | --- |

***THIS IS ONLY FOR CHILDREN WHO ARE AGED BETWEEN 2 AND 12 YEARS OF AGE****.*

*MOTHER/CAREGIVER AND CHILD OR CHILDREN SHOULD BE TOGETHER DURING THE INTERVIEW.*

*USE ONE QUESTIONNAIRE PER CHILD. USE A SEPARATE QUESTIONNAIRE FOR EACH CHILD.*

**____________________________________________________________________________________**

**ID Information**

Mother/caregiver Name: _________________________ __________________________

Family Given

Child Name: _________________________ __________________________

Family Given

Village Name:­­­­­­­­­­­­­­­­­­­­_________________________________________________

**PID**

| **Sub-district ID**  (1 OR 2) | **Village ID** | **RW** | | **RT** | | **House ID** | | | **Individual ID** | |
| --- | --- | --- | --- | --- | --- | --- | --- | --- | --- | --- |
|  |  |  |  |  |  |  |  |  |  |  |

1. **Demographic Information**

***What is the child’s …? (ask if not easily apparent)***

| 1.1 Gender: Male |  | Female |  |  |
| --- | --- | --- | --- | --- |

| \|  \|  \| \| --- \| --- \|  \|  \|  \| \| --- \| --- \|   1.2 Age: years, | months |
| --- | --- | --- | --- | --- | --- |

1.3 Status within household (✓)

| 1) My child |  |  |
| --- | --- | --- |
| 2) Other (not my child)  *Specify:* | | |

1.4 Formal education (✓)

| 1) Attending school |  |
| --- | --- |
| 2) Not attending school |  |

**2. Housing conditions**

2.1 How many people live in this home?

**NOT FOR THE CHILD’S QUESTIONNAIRE**

2.2 What is the approximate area of the house?

**NOT FOR THE CHILD’S QUESTIONNAIRE**

2.3 How much floor space is cement/concrete (dry)? (✓)

**NOT FOR THE CHILD’S QUESTIONNAIRE**

2.4 Which part(s) of the house has a cement/dry floor? (*Multiple responses allowed*) (✓)

**NOT FOR THE CHILD’S QUESTIONNAIRE**

2.5 What are the walls made of? (*Multiple responses allowed*)

**NOT FOR THE CHILD’S QUESTIONNAIRE**

**3. Use of latrines**

3.1 Where does this child usually have a bowel motion? (✓)

| 1) River/bush |  |  |
| --- | --- | --- |
| 2) Public latrines |  |  |
| 3) Neighbours’/relatives’ latrine |  |  |
| 4) Latrine at my home |  |  |
| 5) Other  *Specify:* | | |

3.2 After this child has a bowel motion, how does he/she clean himself/herself? (✓)

| 1) With leaves |  |  |
| --- | --- | --- |
| 2) In the river |  |  |
| 3) With paper In the bathroom |  |  |
| 4) With water in the bathroom |  |  |
| 5) Other  *Specify:* | | |

**4. Water usage**

4.1 Where does this child get water for drinking? (*Multiple responses allowed*) (✓)

|  | ✓ | 4.1.1 If ***YES*** (✓),  how old is it? | 4.1.2 If ***YES*** (✓),  how far is it from a latrine? | |
| --- | --- | --- | --- | --- |
|  |  |  | Distance | No latrine |
| 1) From a public well |  | months | m |  |
| 2) From a household well |  | months | m |  |
| 3) From an artesian source |  | months | m |  |
| 4) From government pipeline (PAM) |  | months | m |  |
| 5) From the river |  | | m |  |
| 6) Buy bottled water |  | | | |
| 7) Other  *Specify:* | | | | |

4.1.3 (If answer to 4.2 was “***Buy bottled water***”) Do you show this child how to check whether the seal on
 the bottle is intact? (✓)

| 1) Yes |  |
| --- | --- |
| 2) No |  |

4.2 Do you boil your water for drinking purposes? (✓)

**NOT FOR THE CHILD’S QUESTIONNAIRE**

4.2.1 (If answer to 4.2 was “***YES***”) How long do you boil water? (✓)

**NOT FOR THE CHILD’S QUESTIONNAIRE**

4.3 Where do you get water to wash your kitchen utensils? (*Multiple responses allowed*) (✓)

**NOT FOR THE CHILD’S QUESTIONNAIRE**

4.4. Baby’s formula: Where do (did) you get water for your baby’s formula?
 (*Multiple responses allowed*) (✓)

**NOT FOR THE CHILD’S QUESTIONNAIRE**

4.4.3 Do (Did) you boil your water for your baby’s formula? (✓)

**NOT FOR THE CHILD’S QUESTIONNAIRE**

4.4.4 (If answer to 4.4.3was “***YES***”) How long do you boil water? (✓)

**NOT FOR THE CHILD’S QUESTIONNAIRE**

4.5 Do you use Chlorine tablets in your water? (✓)

**NOT FOR THE CHILD’S QUESTIONNAIRE**

**5. Animals**

5.1 How many animals do you keep? (*Multiple responses allowed*) (✓)

**NOT FOR THE CHILD’S QUESTIONNAIRE**

5.2 Where are the animals enclosed/caged? (*Multiple responses allowed*) (✓)

**NOT FOR THE CHILD’S QUESTIONNAIRE**

QUESTIONS 5.3 AND 5.4 SHOULD BE ANSWERED BY THE CHILD DIRECTLY, NOT BY THE MOTHER/CAREGIVER

5.3 Can domestic animals transmit disease to people? (✓)

| 1) Yes |  |
| --- | --- |
| 2) No |  |
| 3) Do not know, or no response |  |

5.4 To prevent people getting diseases, which is better? (✓)

| 1) Keep domestic animals in a special enclosure |  |
| --- | --- |
| 2) Let animals roam free in the house |  |
| 3) Do not know, or no response |  |

**6. Gastrointestinal and helminth-related diseases**

6.1 Over the past **3 months**, has this child had a bowel sickness?
 (*Prompt:* such as diarrhea, abdominal pain with diarrhea, dysentery, recurrent typhoid)

| 1) Yes |  | (✓) How many times? |  |  | *⇒ Go to 6.1.1   (and 6.1.3)* |
| --- | --- | --- | --- | --- | --- |
|  | | | | | |
|  | | | | | |
| 2) No |  | (✓) *⇒ Go to 6.2* | | | |

6.1.1 (*If answer to 6.1 was “****Yes****”*) How did you treat the sickness? (*Multiple responses allowed*) (✓)

| 1) Bought medication at a shop |  |  |
| --- | --- | --- |
| 2) Used public health care centre |  |  |
| 3) Went to see a physician |  |  |
| 4) Was hospitalised as in-patient |  |  |
| 5) Do not remember |  |  |
| 6) No treatment |  |  |
| 7) Other  *Specify:* | | |

6.1.2 (***Adults****: If answer to 6.1 was “****Yes****”*) Were you absent from work due to bowel sickness/infection
 in the past 3 months?

**NOT FOR THE CHILD’S QUESTIONNAIRE**

6.1.3 (***Students****: if answer to 6.1 was “****Yes****”*) Was this child absent from school due to bowel
 sickness/infection in the past 3 months?

| 1) Yes |  | (✓) How many days?   \|  \|  \| \| --- \| --- \| |
| --- | --- | --- | --- | --- |
|  | | |
| 2) No |  | (✓) |

QUESTIONS 6.2 THROUGH 6.17 SHOULD BE ANSWERED BY THE CHILD DIRECTLY, NOT BY THE MOTHER/CAREGIVER

6.2 In your opinion, what makes people sick with bowel infections (diarrhea, dysentery, typhoid, etc)?

(*Multiple responses allowed*) (✓)

| 1) Bacteria, or viruses |  |  |
| --- | --- | --- |
| 2) Worms |  |  |
| 3) Do not know, or no response |  |  |
| 4) Witchcraft, or Satan |  |  |
| 5) Other  *Specify:* | | |

6.3 Can you prevent diarrhea by washing your hands before you eat? (✓)

| 1) Yes |  |
| --- | --- |
| 2) No |  |
| 3) Do not know, or no response |  |

6.4 Can you prevent diarrhea by regularly cutting your nails? (✓)

| 1) Yes |  |
| --- | --- |
| 2) No |  |
| 3) Do not know, or no response |  |

6.5 Can you help prevent diarrhea by washing eating utensils or kitchen utensils with clean water (boiled
 water, water from sealed bottles, PAM, or artesian source)? (✓)

| 1) Yes |  |
| --- | --- |
| 2) No |  |
| 3) Do not know, or no response |  |

6.6 Can you help prevent diarrhea by keeping food away from insects? (✓)

| 1) Yes |  |
| --- | --- |
| 2) No |  |
| 3) Do not know, or no response |  |

6.7 Can you help prevent diarrhea by only buying foods that are covered? (✓)

| 1) Yes |  |
| --- | --- |
| 2) No |  |
| 3) Do not know, or no response |  |

6.8 Can you help prevent diarrhea by only drinking water that was boiled? (✓)

| 1) Yes |  |
| --- | --- |
| 2) No |  |
| 3) Do not know, or no response |  |

6.9 Can you name the types of worms that can be found in the human stomach?
 (*Multiple responses allowed*) (✓)

| 1) Pinworms (Oxyuris) |  |  |
| --- | --- | --- |
| 2) Roundworms (Ascaris) |  |  |
| 3) Whipworms (Ancyclostoma) |  |  |
| 4) Hookworms |  |  |
| 5) Tapeworms |  |  |
| 6) Do not know, or no response |  |  |
| 7) Other  *Specify*: | | |

6.10 In your opinion, can worms make you sick? (✓)

| 1) Yes |  |
| --- | --- |
| 2) No |  |
| 3) Do not know, or no response |  |

6.11 What are the symptoms of Roundworm infection (Ascaris)? (*Multiple responses allowed*) (✓)

| 1) Fever and dizziness |  |
| --- | --- |
| 2) Coughing up phlegm (sputum) |  |
| 3) Anaemia |  |
| 4) Quickly becoming exhausted |  |
| 5) Do not know, or no response |  |

6.12 What are the symptoms of Pinworm infection (Oxyuris)? (*Multiple responses allowed*) (✓)

| 1) Itching in the anus |  |
| --- | --- |
| 2) Abdominal pain |  |
| 3) Do not know, or no response |  |

6.13 What are the symptoms of Hookworm infection? (*Multiple responses allowed*) (✓)

| 1) Itching in the anus |  |
| --- | --- |
| 2) Abdominal pain |  |
| 3) Do not know, or no response |  |

6.14 Are bacteria and worm eggs contained in human faeces? (✓)

| 1) Yes |  |
| --- | --- |
| 2) No |  |
| 3) Do not know, or no response |  |

6.15 When people pass motions in the river or bush, do you think it can spread those diseases or worms
 we mentioned above? (✓)

| 1) Yes |  |
| --- | --- |
| 2) No |  |
| 3) Do not know, or no response |  |

6.16 Do you think the faeces of healthy people can also contain those diseases we mentioned above? (✓)

| 1) Yes |  |
| --- | --- |
| 2) No |  |
| 3) Do not know, or no response |  |

6.17 Do you consider that passing a motion in the river or garden is good health behavior or not? (✓)

| 1) Not good |  |
| --- | --- |
| 2) It does not matter |  |
| 3) Good |  |
| 4) Do not know, or no response |  |

QUESTIONS 7.1 THROUGH 7.3 SHOULD BE ANSWERED BY THE CHILD DIRECTLY, NOT BY THE MOTHER/CAREGIVER

**7. Washing hands**

7.1 When do you wash your hands? (*Multiple answers possible)*.
 ***Let the child answer the question and do* not *suggest possible answers.*** ***Place (✓) in the appropriate box below.***

7.1.1 After toilet

| 1) Always |  |  | 2) Often |  |  | 3) Sometimes |  |
| --- | --- | --- | --- | --- | --- | --- | --- |

7.1.2 Before eating

| 1) Always |  |  | 2) Often |  |  | 3) Sometimes |  |
| --- | --- | --- | --- | --- | --- | --- | --- |

7.1.3 After eating

| 1) Always |  |  | 2) Often |  |  | 3) Sometimes |  |
| --- | --- | --- | --- | --- | --- | --- | --- |

7.1.4 Before preparing food

| 1) Always |  |  | 2) Often |  |  | 3) Sometimes |  |
| --- | --- | --- | --- | --- | --- | --- | --- |

7.1.5 After changing diaper

| 1) Always |  |  | 2) Often |  |  | 3) Sometimes |  |
| --- | --- | --- | --- | --- | --- | --- | --- |

7.1.6 When coming home

| 1) Always |  |  | 2) Often |  |  | 3) Sometimes |  |
| --- | --- | --- | --- | --- | --- | --- | --- |

7.1.7 Before prayers

| 1) Always |  |  | 2) Often |  |  | 3) Sometimes |  |
| --- | --- | --- | --- | --- | --- | --- | --- |

7.1.8 Other (Specify:_____________________________________)

| 1) Always |  |  | 2) Often |  |  | 3) Sometimes |  |
| --- | --- | --- | --- | --- | --- | --- | --- |

7.2 How often do you use soap when you wash your hands? (✓)

| 1) Always |  |
| --- | --- |
| 2) Often |  |
| 3) Sometimes |  |
| 4) Never |  |
| 5) No response |  |

7.3 Why would you possibly NOT use soap while washing your hands? (✓)

| 1) Forget |  |
| --- | --- |
| 2) In hurry |  |
| 3) Soap not available |  |
| 4) Habit |  |
| 5) Other  Specify: | |
| 6) No response | |

QUESTIONS 8.1 THROUGH 8.9 SHOULD BE ANSWERED BY THE CHILD DIRECTLY, NOT BY THE MOTHER/CAREGIVER

**8. Behaviour related to gastrointestinal diseases and worms**

8.1 Do you go out into the paddy fields or other fields? (✓)

| 1) Always (*every day*) |  |  |
| --- | --- | --- |
| 2) Often (*once a week*) |  |  |
| 3) Sometimes (*once a month*) |  |  |
| 4) Never |  | *⇒ If* ***never****, go to 8.3* |
| 5) No response |  | *⇒ If* ***no response****, go to 8.3* |

8.2 (***If answered 1)-3) above***) Do you wear shoes/sandals when you go out into the paddy fields or other
 fields? (✓)

| 1) Always |  |
| --- | --- |
| 2) Often |  |
| 3) Sometimes |  |
| 4) Never |  |
| 5) No response |  |

8.3 Do you wash or peel fruit before you eat it? (✓)

| 1) Always |  |
| --- | --- |
| 2) Often |  |
| 3) Sometimes |  |
| 4) Never |  |
| 5) No response |  |

8.4 Do you eat raw or un-boiled vegetables? (✓)

| 1) Always |  |
| --- | --- |
| 2) Often |  |
| 3) Sometimes |  |
| 4) Never |  |
| 5) No response |  |

8.5 Do you eat with a spoon or a similar utensil? (✓)

| 1) Always |  |
| --- | --- |
| 2) Often |  |
| 3) Sometimes |  |
| 4) Never |  |
| 5) No response |  |

8.6 How often do you cut your fingernails? (✓)

| 1) Once in a week or more often |  |
| --- | --- |
| 2) About once in two weeks |  |
| 3) Less often than once in two weeks |  |
| 4) No response |  |

8.7 Do you bite or suck your fingers/fingernails? (✓)

| 1) Always |  |
| --- | --- |
| 2) Often |  |
| 3) Sometimes |  |
| 4) Never |  |
| 5) No response |  |

8.8 Do flies get into your food at home? (✓)

| 1) Always |  |
| --- | --- |
| 2) Often |  |
| 3) Sometimes |  |
| 4) Never |  |
| 5) No response |  |

8.9 Do you buy food from street traders if the food is covered? (✓)

| 1) Always |  |
| --- | --- |
| 2) Often |  |
| 3) Sometimes |  |
| 4) Never |  |
| 5) No response |  |

**9. Items checked during the visit (observations by interviewer)**

9.1 Can you show me your nails? (✓)

| 1) All clean |  |
| --- | --- |
| 2) Some dirty |  |
| 3) All dirty |  |

9.2 Can you show me your hands? (✓)

| 1) Clean |  |
| --- | --- |
| 2) Somewhat dirty |  |
| 3) Very dirty |  |

9.3 Do you have any itching in your anus today? (✓)

| 1) Yes |  |  | 2) No |  |  | 3) No response |  |
| --- | --- | --- | --- | --- | --- | --- | --- |

9.4 Are there worms in your stool today? (✓)

| 1) Yes |  |
| --- | --- |
| 2) No |  |
| 3) Do not know |  |
| 4) No bowel movement today |  |
| 5) No response |  |

9.5 Do you have any stomach/abdominal pain at this time? (✓)

| 1) Yes |  |  | 2) No |  |  | 3) No response |  |
| --- | --- | --- | --- | --- | --- | --- | --- |

------------------------------------------------------------------------------------------------------------------------

**Thank you very much for participating! I have just one more thing I would like to ask you:**

***Can I measure a number of things about you?***

**10. Anthropometric Information**

|  |  |  |
| --- | --- | --- |

|  |  |  |
| --- | --- | --- |

- 1. Height (cm) 10.2 Weight (kg)
  2. Skinfolds (triceps)_____________(Millimetres)

10.4 Hb________________
