# Supplementary material for: Epidemiology of soil-transmitted helminth infections in Semarang, Central Java, Indonesia
Source: PLoS Negl Trop Dis. 2020 Dec 28;14(12):e0008907. doi: 10.1371/journal.pntd.0008907 (PMC7793285; doi:10.1371/journal.pntd.0008907)
Supplement: S1 Table — (DOCX) [file pntd.0008907.s003.docx]

S1Table. Participant and selected household demographics for each surveyed village in Semarang.

| Demographic variable | Village | | | | | | | | | | | | | | | | |
| --- | --- | --- | --- | --- | --- | --- | --- | --- | --- | --- | --- | --- | --- | --- | --- | --- | --- |
|  | Sukorejo  (n=584) | Nongko Sawit  (n=566) | Gunung Pati (n=569) | Plalangan (n=285) | Sumurejo (n=375) | Pakintelan (n=379) | Mangunsari (n=342) | Sekaran (n=413) | Karang Malang (n=284) | Polaman (n=325) | Purwosari (n=328) | Tambangan (n=285) | Jati Barang (n=346) | Kedung Pane (n=562) | Ngadirgo (n=355) | Wonolopo (n=468) | p-value |
| Mean age in years^#^ (SD) | 32.0 (19.3) | 32.7 (20.5) | 34.8 (20.3) | 32.9 (20.2) | 34.3 (19.3) | 31.5 (19.5) | 33.4  (20.5) | 33.7 (18.9) | 37.3 (20.9) | 34.9 (20.9) | 35.5 (20.6) | 31.0  (18.5) | 32.9 (19.0) | 33.7 (19.7) | 32.2 (20.0) | 34.9 (20.0) | 0.002^a^ |
| Gender^#^  Female  Male | 47.4  52.6 | 50.9  49.1 | 50.9  49.1 | 49.8  50.2 | 51.5  48.5 | 51.6  48.4 | 48.2  51.8 | 47.0  53.0 | 49.3  50.7 | 45.8  54.2 | 47.0  53.0 | 50.9  49.1 | 52.9  47.1 | 52.3  47.7 | 49.0  51.0 | 50.9  49.1 | 0.770^b^ |
| Education^#^  Not at sch.  Child at sch.  Elementary  Junior  Senior  College/higher | 5.6  13.7  11.5  27.7  19.8  21.8 | 6.3  14.5  8.8  28.8  20.8  20.8 | 5.5  8.8  13.5  34.6  19.6  18.1 | 5.7  12.2  5.3  37.3  18.6  20.9 | 4.4  11.5  7.4  37.0  21.0  17.8 | 8.3  9.9  9.4  37.8  18.2  16.4 | 6.3  14.0  11.9  32.7  21.4  13.7 | 5.1  11.2  16.3  29.9  17.3  20.2 | 2.2  9.4  17.7  39.7  19.1  11.9 | 8.2  11.0  9.1  37.2  22.4  12.0 | 7.4  10.2  23.8  33.4  16.7  8.4 | 5.0  15.1  12.2  31.7  23.7  12.2 | 7.5  8.1  11.7  30.0  27.6  15.0 | 4.3  14.7  10.4  30.0  21.9  18.7 | 7.9  10.2  15.2  31.8  18.4  16.6 | 5.7  9.8  9.6  33.9  18.3  22.7 | <0.001^b^ |
| Occupation– adult^#^  Student  Private sector  Farmer  Public sector  Self-employed  Unemployed  Home duties  Other/unspecified | 13.1  4.5  0.4  1.7  15.2  10.3  18.2  36.5 | 13.0  9.0  7.4  2.2  14.6  9.0  16.0  28.8 | 10.3  4.3  6.4  0.4  10.1  12.4  17.8  39.5 | 6.7  4.7  2.7  1.3  7.6  3.1  10.8  13.0 | 9.0  5.2  3.4  0.2  9.0  5.4  12.2  21.7 | 11.7  3.4  1.3  0.4  3.8  7.4  10.6  31.2 | 4.3  1.9  2.8  0.0  4.9  14.4  9.2  20.0 | 6.5  15.7  3.6  0.7  10.1  5.8  13.0  21.3 | 4.1  4.7  7.1  0.2  3.2  6.9  10.7  15.7 | 7.0  1.3  7.2  0.0  8.3  5.2  9.9  20.0 | 4.3  1.7  11.2  0.0  4.7  6.4  7.5  21.5 | 7.9  14.2  2.9  1.1  2.5  1.6  9.7  10.8 | 0.4  12.0  3.2  0.2  3.9  23.2  0.4  16.5 | 11.5  14.6  5.6  1.3  14.4  7.2  22.5  25.6 | 7.7  1.1  3.6  0.2  6.0  5.2  7.7  29.0 | 10.8  11.0  7.0  1.8  10.6  6.3  15.1  26.1 | <0.001^b^ |
| Mean no. of household members (SE) | 4.1  (0.12) | 4.0  (0.14) | 3.7 (0.13) | 3.6  (0.13) | 3.6  (0.14) | 3.6  (0.14) | 3.6  (0.16) | 3.6  (0.13) | 3.0  (0.14) | 3.4  (0.14) | 3.4 (0.13) | 3.9  (0.18) | 3.6  (0.13) | 3.7  (0.10) | 3.6  (0.15) | 3.8  (0.13) | <0.001^c^ |
| % of households living below poverty index | 72.2 | 65.0 | 78.9 | 63.3 | 56.1 | 71 | 75.5 | 67.2 | 73.7 | 66.1 | 60.6 | 61.4 | 64.3 | 64.2 | 69.7 | 60.3 | 0.002^bd^ |
| % of households with goats | 2.3 | 4.1 | 0.6 | 11.5 | 8.3 | 9.6 | 5.6 | 11.2 | 10.4 | 2.8 | 4.8 | 7.1 | 11.4 | 8.6 | 6.0 | 7.1 | 0.011^b^ |

^#^ % of individuals in each surveyed village (excluding missing values)

^a^ One-way ANOVA [F(15, 6428) = 2.35, p = 0.002)]. Post-hoc analysis revealed significant differences in mean age between villages: Pakintelan and Karang Malang (p = 0.02); Sukorejo and Karang Malang (p = 0.02) and; Tambangan and Karang Malang (0.02).

^b^ Chi-square test

^c^ One-way ANOVA [F(15, 2139) = 3.12, p<0.001)].

^d^ As defined by the World Bank definition of poverty [1]. Calculated based on the average exchange rate in 2014 [2].

1. The World Bank Group. *Principles and Practice in Measuring Global Poverty*. 2016.

2. Exchange Rates UK. *US Dollar to Indonesian Rupiah Spot Exchange Rates for 2014*. 2019. [Accessed 16 September 2019]; Available from: <https://www.exchangerates.org.uk/USD-IDR-spot-exchange-rates-history-2014.html>.
