# Supplementary material for: Epidemiology of soil-transmitted helminth infections in Semarang, Central Java, Indonesia
Source: PLoS Negl Trop Dis. 2020 Dec 28;14(12):e0008907. doi: 10.1371/journal.pntd.0008907 (PMC7793285; doi:10.1371/journal.pntd.0008907)
Supplement: S2 Table — (DOCX) [file pntd.0008907.s004.docx]

S2 Table. Village level prevalence of soil-transmitted helminths (STH) (% of households with at least one infected member).

| Village | No. of households | STH | | | *A. lumbricoides* | | | *T. trichiura* | | | Hookworm | | |
| --- | --- | --- | --- | --- | --- | --- | --- | --- | --- | --- | --- | --- | --- |
|  |  | **%** | **95% CIs** | **p-value** | **%** | **95% CIs** | **p-value** | **%** | **95% CIs** | **p-value** | **%** | **95% CIs** | **p-value** |
| Gunungpati | 179 | 57.5 | 49.9-64.9 | 0.05 | 21.2 | 15.5-28.0 | <0.001 | 0.0 | - | - | 3.9 | 1.6-7.9 | <0.001 |
| Jati Barang | 117 | 75.2 | 66.4-82.7 | <0.001 | 35.9 | 27.2-45.3 | 0.003 | 0.0 | - | - | 15.4 | 9.4-23.2 | <0.001 |
| Karang Malan | 106 | 64.2 | 54.3-73.2 | 0.005 | 26.4 | 18.3-35.9 | <0.001 | 1.9 | 0.2-6.6 | <0.001 | 7.5 | 3.3-14.3 | <0.001 |
| Kedung Pane | 177 | 62.7 | 55.1-69.9 | <0.001 | 23.2 | 17.2-30.1 | <0.001 | 0.0 | - | - | 15.3 | 10.3-21.4 | <0.001 |
| Mangunsari | 114 | 63.2 | 53.6-72.0 | 0.006 | 28.9 | 20.8-38.2 | <0.001 | 0.0 | - | - | 12.3 | 6.9-19.7 | <0.001 |
| Ngadirgo | 123 | 65.0 | 55.9-73.4 | 0.001 | 28.5 | 20.7-37.3 | <0.001 | 0.0 | - | - | 13.8 | 8.3-21.2 | <0.001 |
| Nongko Sawit | 176 | 72.7 | 65.5-79.2 | <0.001 | 35.8 | 28.7-43.4 | 0.002 | 1.1 | 0.1-4.0 | <0.001 | 9.7 | 5.7-15.0 | <0.001 |
| Pakintelan | 126 | 69.8 | 61.0-77.7 | <0.001 | 35.7 | 27.4-44.7 | 0.002 | 1.6 | 0.2-5.6 | <0.001 | 11.1 | 6.2-17.9 | <0.001 |
| Plalangan | 97 | 58.8 | 48.3-68.7 | 0.104 | 25.8 | 17.4-35.7 | <0.001 | 0.0 | - | - | 9.3 | 4.3-16.9 | <0.001 |
| Polaman | 112 | 77.7 | 68.8-85.0 | <0.001 | 38.4 | 29.4-48.1 | 0.02 | 0.0 | - | - | 11.6 | 6.3-19.0 | <0.001 |
| Purwosari | 132 | 56.8 | 47.9-65.4 | 0.139 | 24.2 | 17.2-32.5 | <0.001 | 0.0 | - | - | 8.3 | 4.2-14.4 | <0.001 |
| Sekaran | 139 | 48.2 | 39.7-56.8 | 0.735 | 0.0 | - | - | 22.3 | 15.7-30.1 | <0.001 | 0.0 | - | - |
| Sukorejo | 179 | 57.5 | 49.9-64.9 | 0.052 | 19.0 | 13.5-25.5 | <0.001 | 0.6 | 0.0-3.1 | <0.001 | 7.8 | 4.3-12.8 | <0.001 |
| Sumurejo | 144 | 61.1 | 52.6-69.1 | 0.01 | 25.7 | 18.8-33.6 | <0.001 | 0.0 | - | - | 7.6 | 3.9-13.3 | <0.001 |
| Tambangan | 91 | 76.9 | 66.9-85.1 | <0.001 | 26.4 | 17.7-36.7 | <0.001 | 1.1 | 0.0-6.0 | <0.001 | 13.2 | 7.0-21.9 | <0.001 |
| Wonolopo | 151 | 58.3 | 50.0-66.2 | 0.05 | 13.2 | 8.3-19.7 | <0.001 | 0.7 | 0.0-3.6 | <0.001 | 18.5 | 12.7-25.7 | <0.001 |
