# Supplementary material for: Epidemiology of soil-transmitted helminth infections in Semarang, Central Java, Indonesia
Source: PLoS Negl Trop Dis. 2020 Dec 28;14(12):e0008907. doi: 10.1371/journal.pntd.0008907 (PMC7793285; doi:10.1371/journal.pntd.0008907)
Supplement: S3 Table — (DOCX) [file pntd.0008907.s005.docx]

S3 Table. STH infection and health measurements for children 12 years and younger

| Variable | No infection  (n = 781) | STH positive  (n = 380) | P-value |
| --- | --- | --- | --- |
| Height (cm) – Mean (SD) | 115.5 (18.0) | 113.9 (16.9) | 0.18^a^ |
| Weight (kg) – Mean (SD) | 22.4 (8.2) | 21.9 (7.7) | 0.19^b^ |
| BMI (n=998) – Mean (SD) | 16.9 (4.4) | 17.0 (4.1) | 0.79^c^ |
| BMI category  Underweight  Normal  Overweight  Obese | n =657  120 (18.3)  373 (56.8)  82 (12.5)  82 (12.5) | n = 306  41 (13.4)  190 (62.1)  32 (10.5)  43 (14.1) | 0.17^d^ |
| Hb (g/L)- Mean (SD) | 12.0 (1.9) | 12.0 (1.9) | 0.87^e^ |
| Anaemia category  Non-anaemic (n, %)  Mild anaemia (n, %)  Moderate anaemia (n, %)  Severe anaemia (n, %) | n = 598  399 (66.7)  65 (10.9)  117 (19.6)  17 (2.8) | n = 290  197 (67.5)  26 (9.0)  60 (20.7)  7 (2.4) | 0.80^d^ |

^a^T-test (t = 1.35, df = 664.3, p = 0.18)

^b^T-test ( t= 1.33, df = 744.9, p = 0.19)

^c^T-test (t= -0.27, df = 652.0, p = 0.79)

^d^Chi-squared test

^e^T-test (t = 0.16, df = 556.6, p = 0.87)
