# Supplementary material for: Epidemiology of soil-transmitted helminth infections in Semarang, Central Java, Indonesia
Source: PLoS Negl Trop Dis. 2020 Dec 28;14(12):e0008907. doi: 10.1371/journal.pntd.0008907 (PMC7793285; doi:10.1371/journal.pntd.0008907)
Supplement: S4 Table — (DOCX) [file pntd.0008907.s006.docx]

S4 Table. Anaemia in surveyed children between 2 and 12 years old.

| Anaemia category | Male  n=465 | Female  n=423 | Non-school aged (2-5 years) n=291 | School aged (6-12 years) n=597 |
| --- | --- | --- | --- | --- |
| Non-anaemic | 320 (69.9) | 276 (65.2) | 206 (70.8) | 390 (65.3) |
| Mild anaemia | 46 (9.9) | 45 (10.6) | 42 (14.4) | 49 (8.2) |
| Moderate anaemia | 83 (17.8) | 94 (22.2) | 39 (13.4) | 138 (23.1) |
| Severe anaemia | 16 (3.4) | 8 (1.9) | 4 (1.4) | 20 (3.4) |
| P-value^a^ | 0.20 | | <0.001 | |

^a^Chi-square test
