# Supplementary material for: Epidemiology of soil-transmitted helminth infections in Semarang, Central Java, Indonesia
Source: PLoS Negl Trop Dis. 2020 Dec 28;14(12):e0008907. doi: 10.1371/journal.pntd.0008907 (PMC7793285; doi:10.1371/journal.pntd.0008907)
Supplement: S5 Table — (DOCX) [file pntd.0008907.s007.docx]

S5 Table. BMI categories adjusted for age and gender

| BMI category* (percentile) | Male  n=520 | Female  n=443 | Total  n = 963 | Non-school aged (2-5 years) n=307 | School aged (6-12 years) n=656 |
| --- | --- | --- | --- | --- | --- |
| Underweight (<5^th^) | 87 (16.7) | 74 (16.7) | 161 (16.7) | 48 (15.6) | 113 (17.2) |
| Normal BMI (5^th^ – 85^th^) | 301 (57.9) | 262 (59.1) | 563 (58.5) | 159 (51.8) | 404 (61.6) |
| Overweight (≥ 85^th^ – 95^th^) | 61 (11.7) | 53 (12.0) | 114 (11.8) | 38 (12.4) | 76 (11.6) |
| Obese (>95^th^) | 71 (13.7) | 54 (12.2) | 125 (13.0) | 62 (20.2) | 63 (9.6) |
| P-value | 0.93 | |  | <0.001 | |

*Terminology based on: Barlow SE and the Expert Committee. Expert committee recommendations regarding the prevention, assessment, and treatment of child and adolescent overweight and obesity: summary report. Pediatrics. 2007;120 (suppl 4):s164-92.
