# Supplementary material for: Epidemiology of soil-transmitted helminth infections in Semarang, Central Java, Indonesia
Source: PLoS Negl Trop Dis. 2020 Dec 28;14(12):e0008907. doi: 10.1371/journal.pntd.0008907 (PMC7793285; doi:10.1371/journal.pntd.0008907)
Supplement: S6 Table — (DOCX) [file pntd.0008907.s008.docx]

S6 Table. Latrine usage and cleaning practices.

| Latrine usage | STH prevalence (%) | P-value |
| --- | --- | --- |
| Latrine present in home (n = 3133) | 33.5 | 0.52 |
| Usual place for bowel motion  Household latrine (n = 3555)  Neighbour/relative’s latrine (n = 1293)  Public latrine (n = 313)  River/bush (n = 1213)  Other (n = 86) | 34.1  35.2  32.6  31.7  39.5 | 0.27^a^ |
| Cleaning practices after bowel motion  Leaves (n = 7)  River water (n = 1174)  Paper in bathroom (n = 27)  Water in bathroom (n = 5240)  Other (n = 7) | 57.1  31.2  29.6  34.4  42.9 | 0.14^b^ |

^a^Chi-squared test

^b^Chi-squared test with simulated p-value
