# Supplementary material for: Epidemiology of soil-transmitted helminth infections in Semarang, Central Java, Indonesia
Source: PLoS Negl Trop Dis. 2020 Dec 28;14(12):e0008907. doi: 10.1371/journal.pntd.0008907 (PMC7793285; doi:10.1371/journal.pntd.0008907)
Supplement: S7 Table — (DOCX) [file pntd.0008907.s009.docx]

S7 Table. Handwashing practices

| Handwashing practices | Infected (n=2191) | | Not Infected (n=4275) | | P-value^a^ |
| --- | --- | --- | --- | --- | --- |
|  | n | % | n | % |  |
| After toilet (missing = 1718)   - Always - Often - Sometimes | 804  705  105 | 49.8  43.7  6.5 | 1556  1384  194 | 49.6  44.2  6.2 | 0.89 |
| Before eating (missing = 225 )   - Always - Often - Sometimes | 1327  758  44 | 60.6  34.6  2.0 | 2499  1485  128 | 58.5  34.7  3.0 | 0.04 |
| After eating (missing = 860)   - Always - Often - Sometimes | 1109  748  60 | 50.6  34.1  2.7 | 2037  1519  133 | 47.6  35.5  3.1 | 0.15 |
| Before food preparation (missing = 3550)   - Always - Often - Sometimes | 609  294  99 | 27.8  13.4  4.5 | 1061  634  219 | 24.8  14.8  5.1 | 0.02 |
| After changing diaper (missing = 5964)   - Always - Often - Sometimes | 64  52  47 | 2.9  2.4  2.1 | 128  122  89 | 3.0  2.9  2.1 | 0.65 |
| When coming home (missing = 1671)   - Always - Often - Sometimes | 814  661  170 | 37.2  30.2  7.8 | 1526  1286  338 | 35.7  30.1  7.9 | 0.78 |
| Before prayers (missing = 1654)   - Always - Often - Sometimes | 1158  438  22 | 52.9  20.0  1.0 | 2295  833  66 | 53.7  19.5  1.5 | 0.19 |

^a^Chi-squared test
