# Supplementary material for: Epidemiology of soil-transmitted helminth infections in Semarang, Central Java, Indonesia
Source: PLoS Negl Trop Dis. 2020 Dec 28;14(12):e0008907. doi: 10.1371/journal.pntd.0008907 (PMC7793285; doi:10.1371/journal.pntd.0008907)
Supplement: S8 Table — (DOCX) [file pntd.0008907.s010.docx]

S8 Table. Behaviour related to gastrointestinal diseases and worms

| Behaviours | STH prevalence  (%) | P-value^a^ |
| --- | --- | --- |
| Go out into paddy or other fields  Daily (n = 447)  Weekly (n = 437)  Monthly (n = 713)  Never (n = 4524) | 41.6  41.2  35.8  32.3 | <0.001 |
| Wear shoes in paddy or other fields  Always (n = 893)  Often (n = 243)  Sometimes (n = 258)  Never (n = 208) | 38.7  39.9  31.4  38.9 | 0.14 |
| Wash or peel fruit prior to eating  Always (n = 3125)  Often (n = 1489)  Sometimes (n = 1350)  Never (n = 181) | 32.5  35.9  34.3  30.4 | 0.09 |
| Eat raw or unboiled vegetables  Always (n = 1630)  Often (n = 955)  Sometimes (n = 3343)  Never (n = 854) | 20.1  35.6  34.2  32.0 | 0.11 |
| Use spoon or other utensil for eating  Always (n = 2975)  Often (n = 2560)  Sometimes (n = 697)  Never (n = 58) | 32.8  35.1  32.9  36.2 | 0.23 |
| Cut fingernails regularly  Once a week or more often (n = 620)  About once a fortnight (n = 323)  Less often than once a fortnight (n = 167) | 32.4  31.3  27.5 | 0.16 |
| Suck or bite fingers/fingernails  Always (n = 31)  Often (n = 130)  Sometimes (n = 1883)  Never (n =4280) | 38.7  33.8  33.7  33.1 | 0.95 |
| Flies get into food at home  Always (n = 73)  Often (n = 319)  Sometimes (n = 2237)  Never (n = 3456) | 30.1  32.9  32.4  34.6 | 0.33 |

^a^Chi-squared test
